# Rapid inundation of southern Florida coastline despite low relative sea-level rise rates during the late-Holocene

**DOI:** 10.1038/s41467-019-11138-4

**Published:** 2019-07-19

**Authors:** Miriam C. Jones, G. Lynn Wingard, Bethany Stackhouse, Katherine Keller, Debra Willard, Marci Marot, Bryan Landacre, Christopher E. Bernhardt

**Affiliations:** 1 U.S. Geological Survey, Reston, VA 20192 USA; 2 Department of Earth and Planetary Sciences, Harvard University, Cambridge, MA 02138 USA; 3 U.S. Geological Survey, St. Petersburg, FL 33701 USA

**Keywords:** Palaeoclimate, Palaeoecology, Wetlands ecology

## Abstract

Sediment cores from Florida Bay, Everglades National Park were examined to determine ecosystem response to relative sea-level rise (RSLR) over the Holocene. High-resolution multiproxy analysis from four sites show freshwater wetlands transitioned to mangrove environments 4–3.6 ka, followed by estuarine environments 3.4–2.8 ka, during a period of enhanced climate variability. We calculate a RSLR rate of 0.67 ± 0.1 mm yr^−1^ between ~4.2–2.8 ka, 4–6 times lower than current rates. Despite low RSLR rates, the rapid mangrove to estuarine transgression was facilitated by a period of prolonged droughts and frequent storms. These findings suggest that with higher and accelerating RSLR today, enhanced climate variability could further hasten the loss of mangrove-lined coastlines, compounded by the reductions in natural flow to the coast caused by water management. Climate variability is nonlinear, and when superimposed on increases in RSLR, can complicate estimated trajectories of coastal inundation for resource management and urban planning.

## Abstract (web summary)

Sea-level rise threatens coastal mangroves, with global consequences for these important blue carbon sinks. Here the authors analyse four Holocene sediment cores from islands in Florida Bay and find that mangroves that comprised the South Florida coastline 4–3000 years ago rapidly transitioned to estuarine conditions, despite low rates of sea-level rise, and propose that their demise was driven by high climate variability.

## Introduction

**Fig. 1:**
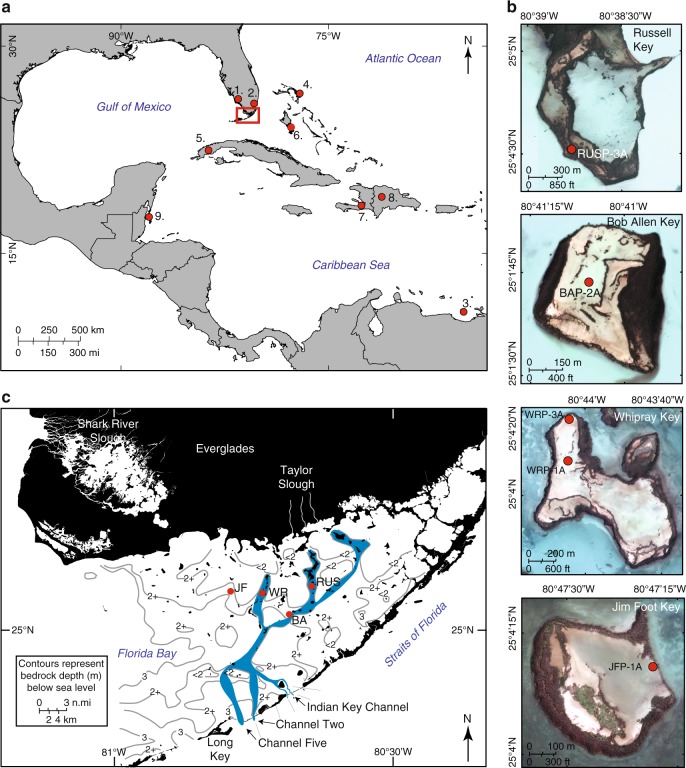
Study area map. **a** Regional map showing the study location (red square) and other sites discussed: (1) Faxahatchee Strand^37^, (2) Northeast Shark River Slough^38^, (3) Cariaco Basin^42^, (4) No Man’s Land, Bahamas^46^, (5) Dos Anas Cave, Cuba^47^, (6) Church’s Bluehole, Andros^48^, (7) Lake Miragoane, Haiti^40^, (8) Valle de Bao, Dominican Republic^50^, (9) Turneffe Atoll, Belize^51^. **b** Aerial photographs show core locations on each island; use of a Russian peat corer eliminated sediment compaction and allowed determination of accurate paleo-elevations of peat deposits. **c** Detail of Florida Bay: locations of the four mud islands studied in Florida Bay, showing bedrock topography and indicating the proposed paleo-drainage channels of Taylor Slough^83^. *Rhizophora* (red) and *Avicennia* (black) mangroves ring the islands, creating a buffer for the fresh to hypersaline carbonate mudflats in the island centers, which were situated below sea level when cores were taken in 2014. Satellite images collected by DigitalGlobe’s WorldView-3 sensor on September 13, 2017

The Holocene environmental history of south Florida has been governed by the interplay of rising sea level, climate variability, and the underlying stable carbonate platform. The rise of global sea level to its near-modern position and its slowdown to 2.1 ± 0.3 mm yr^−1^ between ~8–4 ka and ~0.5–1 mm yr^−1^ occurred during the late Holocene (< 4.2 ka)^1^. This late-Holocene stabilization in the rate of sea-level change is associated with the global expansion of mangrove forests^2^ and other peat and sediment accreting coastal wetlands^3^. Previous studies showed that rising sea level and a change in regional climate allowed for the initiation of freshwater wetlands on the stable Pleistocene carbonate platform^4,5^ in much of the Florida Everglades^5^, including what is now Florida Bay ~5–4 ka^4,6^. Previous studies from Florida Bay and Ten Thousand Islands broadly show a transition to mangrove and estuarine environments ~4–3 ka^4,6^ at a time when the rate of sea-level rise was slowing, but low dating resolution on continuous core records prevented careful examination of rates, nature, and drivers of these transitions. Despite relatively low late-Holocene (< 4.2 ka) rates of RLSR and expansion of coastal wetlands^2,3^ as modern shorelines were being established, the southern shoreline of Florida exhibited continued contraction^6–9^. Here, we apply a multiproxy approach from sediment and peat cores from four central Florida Bay islands (Fig. 1) to examine how climate and sea-level change influenced the mid-to-late Holocene shoreline of south Florida.

Mangroves accumulating autogenic peat on stable carbonate platforms have generally kept pace with SLR over the Holocene^10–12^, including the portion of the early Holocene when the SLR rates were 6–7 mm yr^−1^ (refs 1, 10). These data suggest that mangrove ecosystems are capable of keeping pace with high, and even accelerating, rates of SLR^13,14^. However, studies of mangrove ecosystem response to climate and weather perturbations such as droughts^15^ and hurricanes^16–19^ indicate long-term impacts that lead to shifts in, or loss of, plant communities^16^^,^. Using multiproxy analysis (Supplementary Note 1) from four islands in Florida Bay (western-most Bob Allen Key (BA), Jim Foot Key (JF), Russell Key (RUS), and Buttonwood #7 Key (WR); Fig. 1), we examine the timing, rate and nature of paleoenvironmental change in Florida Bay during the last 5 ky. Specifically, we determine the timing of transitions from freshwater marsh to mangrove (FMT) and mangrove to estuarine (MET) environments using pollen, stable carbon isotope and nitrogen isotope, and molluscan analyses from four well-dated (*n* = 8–10 ^14^C dates/~2.5 -m core) cores in central Florida Bay. We show that the MET occurs during a period of high climate variability, suggesting that transitions between droughts and storms sufficiently stressed the mangrove ecosystems, limiting their ability to keep pace with RSLR.

## Results

### Proxy evidence for Holocene environmental change

**Fig. 2:**
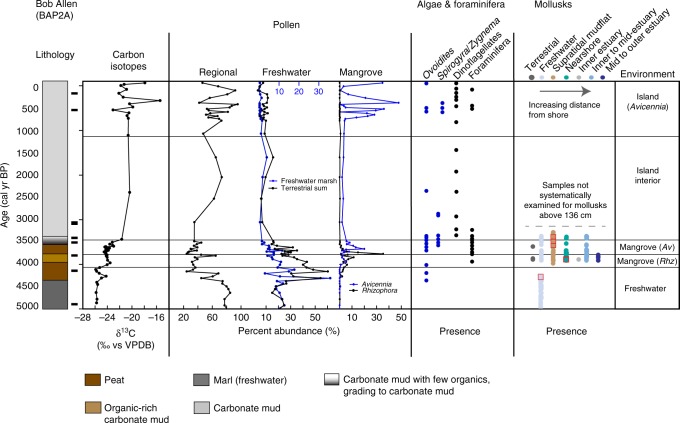
Core summary diagram for Bob Allen Key (BA). Carbon isotopes, selected pollen groups, and presence of algal cysts, foraminifera and dinoflagellates, and mollusks. Regional pollen includes *Pinus* (pine) and *Quercus* (oak). Freshwater marsh sums include Poaceae, Cyperaceae, *Nymphaea, Typha, Rhynchus, Cladium, Schoenoplectus, Sagittaria, Crinum*, and Amaryllidaceae. Terrestrial sum includes all taxa except the regional taxa and mangrove taxa, as well as *Batis maritima* and Amaranthaceae. Presence of certain taxa include *Ovoidites*, *Spirogyra* (closed blue circles); dinoflagellates, foraminifera (black closed circles). Mollusk presence is shown in order of increasing salt-water/estuarine habitat and distance from shoreline from left to right. Red squares indicate abundance or dominance in a given sample. Radiocarbon age control shown by black rectangle next to the lithology

**Fig. 3:**
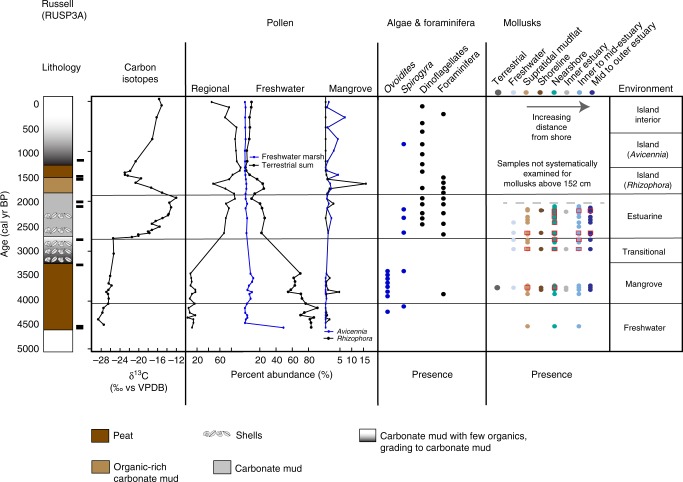
Core summary diagram for Russell Key (RUS). Carbon isotopes, selected pollen groups, and presence of algal cysts, foraminifera and dinoflagellates, and mollusks. Regional pollen includes *Pinus* (pine) and *Quercus* (oak). Freshwater marsh sums include Poaceae, Cyperaceae, *Nymphaea, Typha, Rhynchus, Cladium, Schoenoplectus, Sagittaria, Crinum*, and Amaryllidaceae. Terrestrial sum includes all taxa, except the regional taxa and mangrove taxa, as well as *Batis maritima* and Amaranthaceae. Presence of certain taxa include *Ovoidites*, *Spirogyra* (closed blue circles); dinoflagellates, foraminifera (black closed circles). Mollusk presence is shown in order of increasing salt-water/estuarine habitat and distance from shoreline from left to right. Red squares indicate abundance or dominance in a given sample. Radiocarbon age control shown by black rectangle next to the lithology

**Fig. 4:**
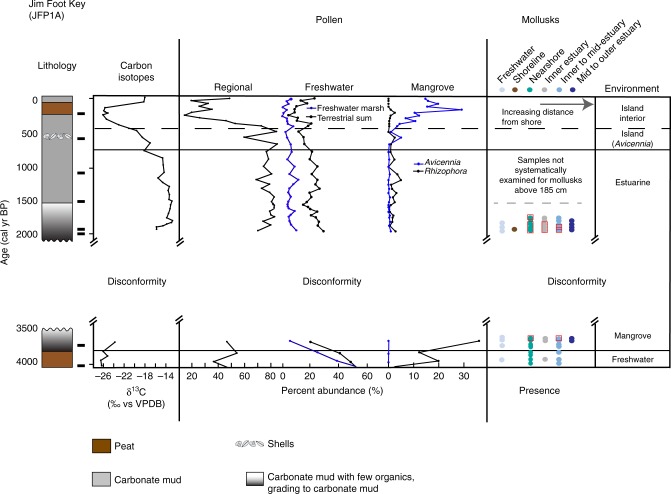
Core summary diagram for Jim Foot (JF) Carbon isotopes, selected pollen groups, and presence of mollusks. Regional pollen includes *Pinus* (pine) and *Quercus* (oak). Freshwater marsh sums include Poaceae, Cyperaceae, *Nymphaea, Typha, Rhynchus, Cladium, Schoenoplectus, Sagittaria, Crinum*, and Amaryllidaceae. Terrestrial sum includes all taxa, except the regional taxa and mangrove taxa, as well as *Batis maritima* and Amaranthaceae. Mollusk presence is shown in order of increasing salt-water/estuarine habitat and distance from shoreline from left to right. Red squares indicate abundance or dominance in a given sample. Radiocarbon age control shown by black rectangle next to the lithology

**Fig. 5:**
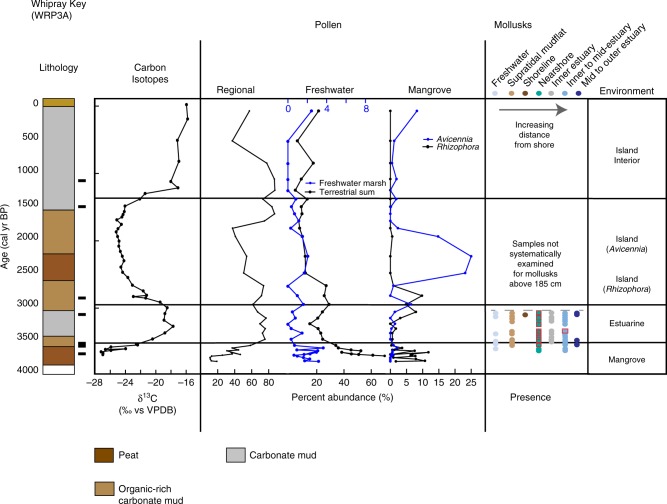
Core summary diagram for Buttonwood #7 Key (WR). Carbon isotopes, selected pollen groups, and presence of mollusks. Regional pollen includes *Pinus* (pine) and *Quercus* (oak). Freshwater marsh sums include Poaceae, Cyperaceae, *Nymphaea, Typha, Rhynchus, Cladium, Schoenoplectus, Sagittaria, Crinum*, and Amaryllidaceae. Terrestrial sum includes all taxa, except the regional taxa and mangrove taxa, as well as *Batis maritima* and Amaranthaceae. Mollusk presence is shown in order of increasing salt-water/estuarine habitat and distance from shoreline from left to right. Red squares indicate abundance or dominance in a given sample. Radiocarbon age control shown by black rectangle next to the lithology

Marl-precipitating marshes, similar to those in the southern Everglades, occupied BA as early as ~5.2 ka (Supplementary Table 1, Supplementary Fig. 1), transitioning to freshwater marsh peat ~4.4 ka (Fig. 2). Peat initiated at RUS ~4.6 ka, with modern analog vegetation corresponding to tree islands that transitioned to sawgrass marshes or sloughs by ~4.2 ka (Fig. 3; Supplementary Note 1, Supplementary Figs 1, 2a; Supplementary Data 1), and JF peat initiated ~4 ka as a sawgrass marsh before transitioning to a mangrove peat ~3.7 ka (Fig. 4), with WR initiating as a freshwater marsh 3.7 ka before transitioning to a mangrove environment soon thereafter (Fig. 5). In each of these cores, the *δ*^13^C signature of the organic fraction of sediment during this freshwater phase ranged from −28 to −25 ‰, consistent with C_3_ plants that occupy the freshwater Florida Everglades^20,21^ (Supplementary Note 1; Supplementary Fig. 2; Supplementary Data 2). Despite the presence of these freshwater indicators, this zone has a presence of nearshore and/or mudflat mollusks in all cores, except BA (Fig. 2; Supplementary Note 1; Supplementary Fig. 3; Supplementary Data 3). At BA, only freshwater gastropods are present from ~5.1 to 4.4 ka in the calcareous marl; faunal indication of proximity to the shoreline does not begin at BA until ~4.0 ka, which agrees with pollen data indicative of a mangrove environment. Apparent rates of peat accumulation for the freshwater peats ranged from 0.21 to 0.4 mm yr^−1^.

**Fig. 6:**
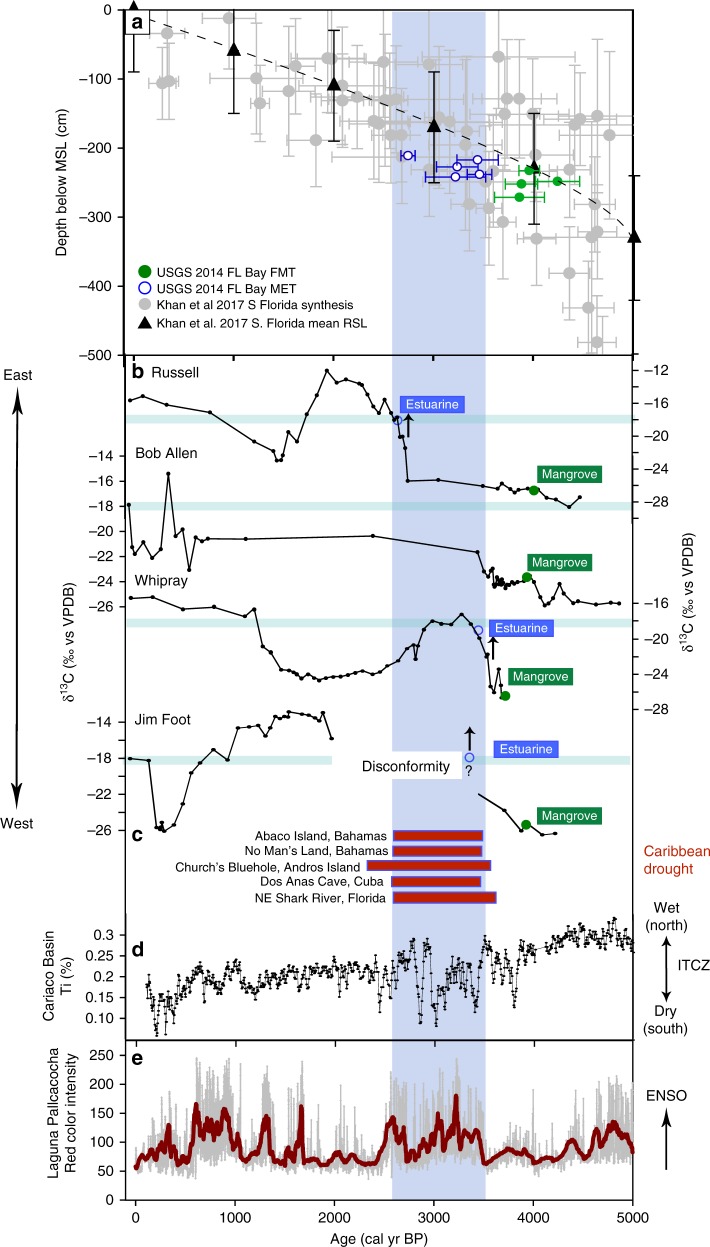
Data summary of regional sea level, environmental transitions, and regional Holocene climate parameters. **a** Mangrove and coral-based sea-level curve synthesis points (gray circles) and mean RSLR curve (black triangles) with error bars^1^. Florida Bay cores are shown in green (FMT) and blue (MET), with age errors. Depth errors are smaller than the dots, and do not account for shallow compaction. Transitions from freshwater peat to mangrove peat (FMT) determined by pollen assemblages, and the mangrove peat to estuarine carbonate mud (MET) determined from pollen and carbon isotope analyses from the four cores (this study) are placed at depth below mean sea level, relative to their 2014 position. FMT   freshwater to mangrove transition, MET   mangrove to estuarine transition. **b** *δ*^13^C for each of the studied cores. Transitions from values < −22‰ to > −18‰ correspond to transitions from C_3_ freshwater and mangrove peats to C_4_-like sea grasses and algae, indicating the shift to estuarine conditions. This isotopic shift is marked by the blue bar. **c** Greater Caribbean sites indicating drought: Abaco island aridity^46^, No Man’s Land, Abaco Island sink hole marine sapropel^45^, Dos Anas Cave *δ*^18^O speleothem record hiatus^47^, Northeast Shark River Slough gypsum occurrence^36^. **d** Titanium recorded in Cariaco Basin, Venezuela sediments, as a proxy for terrestrial runoff^41^, interpreted as a record of ITCZ position. **e** Laguna Pallaconcha lake record of red color intensity^41^, interpreted as a record of El Niño prevalence. High-amplitude variability between anomalously low and high Ti values from ~3.7 to 2.7 ka corresponds to a period of a highly variable ENSO and its coupling to the ITCZ. Light-blue shaded band highlights period of transgression

Although the lithology largely remained peat and the *δ*^13^C indicated plants using C_3_ photosynthesis, pollen analysis indicates that freshwater marshes transitioned to mangrove environments (FMT) between 4.2 and 3.7 ka (Table 1; Figs 2–6). Pollen assemblages after the FMT are most closely aligned with modern dwarf mangrove stands in WR, while JF and BA, and the western and southernmost sites, respectively, have pollen assemblages that most closely resemble the modern southwestern mangrove forest, near the mouth of Shark River. RUS, despite low percentages of *Rhizophora* pollen, has an assemblage that most resembles modern sawgrass marshes and mangroves forests in southwest Florida^22^ (SI). These mangrove zones in all cores have mollusks generally common to habitats ranging from supratidal mudflats to inner to mid-estuary environments. They also contain pollen and algal spore taxa common to freshwater environments (e.g., *Ovoidites, Typha*, *Nymphaea*), as well as gastropods indicative of proximity to freshwater (Hydrobiidae).

**Table 1:** Elevation and sedimentation rates from cores collected on Bob Allen Key (BAP2A), Jim Foot Key (JFP1A), Buttonwood #7 Key (WRP3A), and Russell Bank (RUSP3A)

Core name	2014 surface elevation at core location relative to mean sea level (cm)	Timing of MET (cal years BP)	Transition time (years)	Depth below 2014 SL (cm)	Mangrove sedimentation rate (mm yr^−1^)	Sedimentation rate prior to 15–35% compression (mm yr^−1^)
BAP2A	−35.6	N/A	N/A		1.2	1.62–1.84
JFP1A	−23.1	< 3220- > 2000	Hiatus	−242.1	1	1.15–1.35
WRP3A	−2.2	3460	186–197	−238.4	1.24	1.67–1.90
RUSP3A	−26.2	2744	136	−211.2	0.234	0.32–0.36

Surface elevations were measured using dGPS and measuring depth from bedrock on each island. Timing of inundation and sedimentation rates were identified using age models and centimeter-scale accretion between dated intervals, as described in supplemental information

**Fig. 7:**
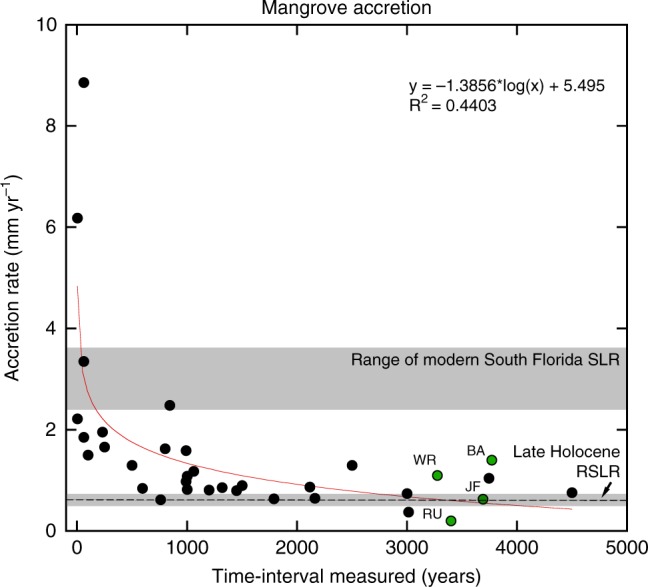
Synthesis of mangrove accretion rates (black dots) across the Caribbean over the last 5.0 ka. Supplementary Table 3 lists contributing data. Red curve is a logarithmic relationship of accretion rates with time since deposition. Dashed line shows the rate of relative sea-level rise in central Florida Bay 3.7–2.8 ka calculated from the studied cores (shown in green). Accretion rate ages are the mean of the measured time period for which the rates were calculated. Lower gray bar indicates range of sea-level rise data based on a south Florida synthesis^1^. Higher gray bar indicates range of relative sea-level rise trends from tide gauge stations at Key West (2.42 mm yr^−1^; 1913–2018) and Vaca Key (3.66 mm yr^−1^; 1971–2018)^34,35^

The mangrove to open-water estuarine transition (MET) in this study was identified using the lithologic transition from peat to carbonate mud and by shifts in organic δ^13^C values from < −22 ‰ (terrestrial plants) to > −18‰ (estuarine sea grasses and marine algae)^20,21^ (Supplementary Information; Supplementary Data 2). Isotope, pollen, and molluscan assemblage analyses (Figs 2–5, Supplementary Data 1–3, Supplementary Figs 2, 3) reveal that three cores (JF, RUS, WR) transgressed to an estuarine environment between 3.4 and  ≥ 2 ka (Figs 3–6), as exemplified not only by the *δ*^13^C, but also a shift away from wetland taxa and toward a more regional pollen assemblage, indicative of estuarine deposition^22^. The exact timing of the MET on JF cannot be determined because of a disconformity between 3.5 and ~2 ka, indicating erosion of the sediment, but the last measured sample prior to the disconformity has pollen assemblages with modern analogs in the southwest mangrove forest, near the mouth of Shark River Slough and C_3_-type *δ*^13^C signatures, while the overlying sample shows a more regional pollen assemblage, consistent with other estuarine cores and a C_4_-like *δ*^13^C signature. Both WR and RUS transitioned in < 200 years, according to their respective age models and shift from C_3_ to C_4_ *δ*^13^C (Table 1; Fig. 6). The BA site transitioned directly from mangrove to mudflat ~3.4 ka, as determined by its pollen and mollusk assemblages and the lack of a transition to a C_4_-like *δ*^13^C signature that defines the estuarine transition in the other cores (Figs 2, 6). This agrees with a previous description of Bob Allen (west) as exclusively supratidal^9^. This transition at BA was concomitant with submergence of WR and within the timeframe of submergence at JF, indicating SLR was less than the accretion rate at the BA site (1.2 mm yr^−1^), despite comparable accretion rates at WR and JF (1–1.24 mm yr^−1^; Fig. 7; Table 1).

### Sea-level determination

We calculated a rate of RSLR by placing the timing and elevation (core depth) of the FMT and MET from the studied cores relative to modern sea level based on measured core-top position relative to modern sea level using differential GPS (Fig. 6a). Using this method, we derive a RSLR rate between 4.2 to 2.7 ka of 0.67 ± 0.1 mm yr^−1^. Because the rate of accretion at BA, which never transitioned to an estuarine environment, at the time was 1.2 mm yr^−1^, the maximum rate of RSLR is capped below the accretion rate at BA. Bulk densities in the lower mangrove peats are 15–35% higher than mangrove peats from cores in Biscayne Bay^23^, suggesting that peat compaction could have occurred (Supplementary Table 2). Factoring a compaction of 15–35% for mangrove peats (Table 1; Fig. 3; Supplementary Note 2; Supplementary Table 2), which have been calculated from the difference between surface to deep-peat organic matter bulk densities^23,24^, indicating the rate of RSLR could not have been higher than 1.4–1.6 mm yr^−1^.

## Discussion

The rate of eustatic sea-level change in the Holocene is associated with northern hemisphere deglaciation, which decreased following the final collapse of the Laurentide ice sheet after 7 ka^25^. Subsequent glacioisostatic adjustments (GIA) related to the forebulge collapse have been modeled^26,27^ and confirmed with relative sea-level curves^1,7,26^, and the effects of GIA are shown to attenuate with distance from the former Laurentide ice sheet margin. Over the period represented by the cores (< 5 ka), GIA in south Florida transitions from a rate of 2.1 ± 0.3 mm yr^−1^ to to 0.6 ± 0.3 mm yr^−1^ during the late-Holocene (< 4.2 ka)^1^, a rate that is confirmed by GIA models^27^. Because the position of peat-forming mangroves is constrained to the upper half of the intertidal zone (e.g., 28,29), their use as sea-level markers has been widely applied (e.g., 1,7). Numerous studies have also shown that under periods of lower rates of sea-level rise, their accretion rates slow, while under higher rates of SLR, accretion rates increase, given the increased accommodation space and their ability to increase belowground biomass through root production and entrapment of organic debris^10,11,14^. While late-Holocene GIA in south Florida is constrained by multiple lines of evidence^1,26,27^, other factors contributing to an increase in the RSLR above which mangroves would have been able to keep pace include an increase in the tidal amplitude or tectonics, but neither of these was a factor over the last 5 ka in south Florida, and no further evidence of a sea-level highstand exists at that time^1^.

By 4.2–3.8 ka, the south Florida coastline reached the central Florida Bay study sites, and as sea level rose at a rate (2.1 slowing to 0.6 ± 0.3 mm yr^−1^)^1^ that outpaced freshwater peat accretion rates (0.2–0.4 mm yr^−1^), all sites transgressed to mangrove environments. Sea level continued to rise at a rate of 0.67 ± 0.1 mm yr^−1^, as calculated by the position and timing of the FMT and MET (Fig. 6a), a rate consistent with other regional sea-level studies^1,7^. Similarly, accretion rates of a brackish marsh with scrub mangrove trees in the Shark River Estuary show accretion rates of 0.66 ± 0.12 mm yr^−1^ that never transgressed to estuarine environments between 3.4 and 2.8ka^30^, which further supports a rate of RSLR in this region of less than ~0.67 mm yr^−1^. Multiple lines of evidence suggest that sea level rose more slowly across the MET than submergence of these peats would suggest. Based on δ^13^C values, which reflect a change in the source material of the organic fraction of the sediment from C_3_ terrestrial and mangrove plants to C_4_-like sea grasses and marine algae, MET transgression was rapid at the two sites (WR, RUS) where this could be measured (< 200 years), despite evidence for pre-inundation rates of accretion higher than the rate of RSLR (Fig. 7; Table 1). Further evidence for a regression of sea level relative to these sites include transitions from *Rhizophora* (low intertidal zone; fringe) to *Avicennia* (high intertidal zone; basin, landward of the fringe) at BA and WR and, to a lesser degree, occurrence of freshwater marshes after the *Rhizophora* peak at RUS, at the time when the southern Florida coastline receded (~3.4 ka to > 2 ka; Figs 2, 3, 5; Supplementary Fig. 2). These data demonstrate that although mangrove peats accreted under some of the lowest rates of SLR of the Holocene, the rapid shift from mangrove to estuarine environment suggests RSLR alone cannot explain the Florida Bay transgression.

Despite these low rates of sea-level rise 3.4 to > ~2 ka, mangrove peat accretion was not able to outpace RSLR and these sites, and with the exception of BA, transgressed to the estuarine environments that comprise much of central Florida Bay today (Figs 2–6). This lies in contrast to the persistence of peat accumulation in the Shark River estuary^30,31^ and across the Caribbean^10,32,33^ (Fig. 7; Supplementary Table 3), indicating that mangroves can keep pace with higher rates of sea-level rise and that other factors contributed to their demise in central Florida Bay from 3.4 to 2.8 ka. Analysis of mangrove accretion rates from south Florida and the greater Caribbean shows a strong temporal relationship toward lower apparent accretion with time since deposition, where a large decline in short-term (annual to decadal) to long-term (millennial) observed accretion occurs (Fig. 7), followed by a stabilization of the observed accretion rate. The logarithmic relationship shown in Fig. 7 suggests that peat accumulation rates at the time of deposition could have been as high as 3.7 to 4.2 mm yr^−1^ 10 years after deposition and 2.5 to 2.75 mm yr^−1^ 100 years after deposition prior to compaction, if decomposition and compaction are the only controls on the logarithmic decline in peat accretion. Compaction is likely to have occurred given the higher bulk densities of the mangrove peats in these cores^23^ (Supplementary Table 2). This compaction occurred either as a result of subsequent overburden of sediment and water, or as a result of unfavorable peat-accreting climatic conditions when these peats were at the surface 3.4 to 2.8 ka. Alternatively, the relationship suggests that mangroves in the late Holocene maintained accretion rates comparable with the rate of SLR and that the most recent increase in accretion is a response to the increase in rates of RLSR in the last ~100 years^34,35^, providing further evidence that mangrove accretion rates can increase with changing rates of SLR.

Because mangrove ecosystems adjust accretion rates by increasing belowground biomass in response to changes in SLR, they are thought to be among the more resilient coastal ecosystems under the right conditions^10,11^. Allogenically driven mangrove accumulation rates are typically higher than mangrove environments that accumulate peat autogenically, but both have been shown to keep pace with sea-level rise^11^. Examples from other sediment-starved basins show mangroves continuously accumulating peat over the last 8000 years, including over periods when the rate of SLR was at least five times higher than late-Holocene rates^10,33^ (Fig. 7). Because autogenic mangrove peat accretion relies on the trees at the surface to be healthy, stress to the mangrove trees themselves, in addition to factors that can reduce peat volume, can impact accretion. Peats are composed primarily of partially decayed organic remains of roots and sometimes other biomass (algal mats, leaves, sea grasses, etc.), making them susceptible to oxidation, root decomposition, shrinkage, erosion, and compaction and root-zone collapse that can affect peat volume, decreasing surface elevations relative to sea level^11^. Factors leading to these processes include droughts and storms. Aboveground productivity of the mangrove trees themselves also likely is a factor controlling long-term carbon storage. A global analysis of modern mangrove growth and height found that warm temperatures, ample rainfall, and frequency of cyclones (with lower cyclones tied to higher mangrove biomass) explained 74% of the variance in global mangrove canopy height^36^.

During the time of peat initiation, results from this study show vegetation assemblages indicative of increased freshwater flow and longer hydroperiods after 4.4 ka, consistent with other studies^5,30,37^. The presence of freshwater marshes prior to ~3.8 ka in what is now central Florida Bay indicates that the studied sites were an extension of the Florida Everglades^6–9^ and that the south Florida shoreline extended at least 15–20 km south of its current position ~5–4 ka, when sea level was 2.5 m lower^1^ (Fig. 2). The following period of 4-~2.8 ka is transitional between mean hydrologic states, from a dry to wet environment in the southwestern Everglades^37^ and wet to dry in the southeastern Everglades^38^, with both locations showing intervals punctuated with wet and dry conditions^37,38^. The east–west directionality of precipitation across the Florida Everglades after 3 ka suggests potential spatial variability, with wetter conditions in the mixed prairie pinelands and cypress forest until 2 ka^36^ and a shift to moderate^39^ and shorter hydroperiods^38^ in the Northeast Shark River Slough after 3–2.8 ka. This shift in precipitation represents a fundamental hydrologic shift in the Florida Everglades^5^ that manifests as a change in the mean state prior to 4 ka to after ~2.5 ka, with a highly variable transitional climatic period in between.

Multiple lines of evidence support the idea that this transitional climate between ~4 and 2.8 ka was a period of high climate variability in the Caribbean^40^ and south Florida (Fig. 6), and is related to a shift in the position of the intertropical convergence zone (ITCZ)^41^ and an increase in El Nino Southern Oscillation (ENSO)^42^. Comparison of the Palmer Drought Severity Index for south Florida with the titanium record from the Cariaco Basin, which is interpreted as reflecting changes in the position of the ITCZ^42^, shows that drought in south Florida is associated with a southward shift of the ITCZ^39,43^. This period of the Holocene shows high-amplitude shifts in the north–south position of the ITCZ, suggesting wet-drought cycles, on multi-centennial timescales^42^ (Fig. 3a). Multiple wet pulses are recorded in a vegetation record from Fakahatchee Strand in southwestern Florida between 4.2 and 2.8 ka, and are interpreted as being associated with ENSO intensification, which increases winter precipitation in south Florida^37^, while gypsum, an evaporative salt, is abundant in an otherwise long-hydroperiod marsh in Northeast Shark River Slough 3.5 to ~3 ka, suggesting intermittent drying prior to its transition to a low-hydroperiod marl-precipitating sawgrass marsh^38^. Additional evidence of drier conditions during the MET (3.5–2.8 ka), include a shift to moderate to short hydroperiods in central Florida^44^, plant assemblages associated with drier environments from a ridge core in the Everglades ridge and slough landscape^45^, and a period of tree island initiation and expansion that has been linked to periods of aridity^43^. More broadly, aridity across the Caribbean is documented ~3.3–2.5 ka, including in the Bahamas^46^, Cuba^47^, Andros Island^48^, Saint Martin^49^, and the Dominican Republic^50^, while in Belize a period of drought from ~4.2–3.5 ka is followed by shift toward higher precipitation 3.3–2 ka^51^ (Fig. 6). This aridity has been attributed to a westward or southward expansion of the North Atlantic Subtropical High, in conjunction with the southward movement of the ITCZ^46^, a pattern that increases the likelihood of extreme rainfall events and droughts over the southeastern North America^52^. Coupled ENSO and ITCZ intensity and position would have produced anomalously wet (dry) conditions and winter cyclones (tropical storms) in south Florida during El Niño (La Niña) phases^53^ and a northward (southward) ITCZ, respectively, conditions that would have amplified the wet-dry effects of each component by itself. Decreased mangrove peat volume may occur during disturbances such as droughts^15^, which was observed during recent ENSO events in Micronesia, as the growth and primary production of mangroves are strongly linked to climate variables, such as temperature, precipitation, evapotranspiration, and solar radiation^16,17^. Drought can also impact mangroves, as salt accumulation increases with stomatal regulation, reducing leaf cell metabolism and carbon assimilation capacity^54^ and lack of sufficient rainfall impacts mangrove productivity^36^. Periods of droughts would have decreased freshwater flux from Taylor Slough, increasing the salinity in Florida Bay, as recorded from lower outflow related to water management^55^.

An analysis of sea-level change and Holocene precipitation variability revealed that a decrease in peat initiation 4.2 to 3.8 ka is related to cooler north Atlantic conditions that resulted in drier conditions in south Florida, while the subsequent increase in peat initiation 3.5 to 1.5 ka in the Greater Everglades is associated with an increase in the regional water balance rather than changes in sea level alone^56^. Peat initiation is associated with highly variable annual to centennial-scale precipitation in south Florida that occurs in relation to ENSO, Atlantic Multidecadal Oscillation (AMO), and the Pacific Decadal Oscillation (PDO) mean state shifts^56^. Other studies have also found the importance of climate variability for the nonlinearity of peatland development processes^56,57^. El Niños in south Florida are commonly associated with prolonged winter storms, which produce persistent winds that can temporarily increase sea levels^58^ and can play a significant role in shoreline erosion and transport of sediment and saline water inland via creeks^18^. Modern patterns of climate variability also show a strong relationship between the Pacific North American index (PNA) and ENSO, whereby + PNA is correlated with El Niño events^53^. Not only does + PNA correspond with wetter winter conditions in south Florida^53^, it also increases the likelihood of freezes in Florida^59^, to which mangroves are intolerant^2,60^, resulting in plant stress and mortality and increasing susceptibility to elevation loss.

In addition to the prolonged droughts across much of the proximate Caribbean, storms, particularly strong hurricanes, may have also contributed to the demise of these coastal mangroves 3.4–2.8 ka. While proxies from our cores show shifts primarily associated with changes in sea level rather than climate across this transition, mollusk assemblages generally point to overwashing of estuarine mollusks into freshwater and mangrove peats, while the presence of gastropods indicative of proximity to freshwater (Hydrobiidae), freshwater algae (*Ovoidites*) and other freshwater marsh taxa (*Typha*, *Nymphaea*) in the mangrove peats indicates freshwater flooding reached these sites, data consistent with storm events. Despite the persistence of El Niño events, which generally suppress Atlantic tropical cyclones^61^, hurricane deposits are frequently recorded in Saint Martin and Charlotte Harbor, Florida from 3.4 to 2.8 ka^49,62^, and in our cores evidence for storm deposits includes estuarine mollusks deposited within the freshwater and mangrove peats and a layer of shells on top of the basal peats in RUS and JF, where an erosional disconformity also exists. Storm deposit evidence from Saint Martin to the south of Florida and Charlotte Harbor to the north of Florida Bay, may suggest a dominant storm trajectory from south to north across the Florida Peninsula. A study from a marl prairie in the Florida Everglades documented increased minerogenic material with a west African provenance, which was suggested to indicate increased tropical cyclone activity in South Florida 4.2–2.8 ka^38^.

In present-day south Florida, tropical storms, particularly category 3 and higher hurricanes, can have a significant impact on the coastline^18,19,63–65^. Studies of the southwest coastal mangrove forests of the Everglades following hurricanes between 1935 and 2005 have documented substantial mangrove die-offs that contribute to increased erosion^16,17,19^ and peat collapse^66^, converting many forested areas to marine environments^16–18,67^. These die-offs are related to many factors, including toppling of trees, denuding of leaves that leads to long-term stress, and depositing of thick storm deposits. Storm surges can deposit thick layers of sediments interior to the coast (5–10 cm documented from Hurricane Donna^63^) and vertical accretion from hurricane deposition has been estimated to be 8 to 17 times greater than the fifty-year averaged annual accretion rate^19^. While deposition of sediments can be beneficial to coastal build-up, the sediments can have a negative impact on black mangroves (*Avicennia*) by smothering the pneumatophores and can lead to die-offs, especially when combined with stress from wind damage^16^. Significant mangrove die-off in a coastal forest can lead to overstepping of the coastal margins^17,18^ by initiating a complex set of feedback loops, including increases in soil temperatures due to loss of the canopy and large depressions created by the downed trees. These changes affect the ability of mangrove saplings to take root, lead to decreases in peat elevation, and additional saltwater intrusion, which ultimately can lead to conversion of the mangrove forest to a mudflat^17,18,67^. Over succeeding years, a steady decline in surface elevation may occur in part due to increased oxidation of the peats and the absence of new growth^66^. These effects from a single storm can be compounded by multiple storms in close succession to one another, with the first storm preconditioning the coast^63^ making it more likely the coastal margin will be overstepped and the mudflats converted to shallow estuarine habitat. Coastal overstepping due to storm activity is a possible contributing mechanism explaining the rapid transgression of the coast between 3.4 and 2.8 ka. Furthermore, a global analysis of mangrove forest height was closely linked to an absence of cyclones, in addition to abundance rainfall and warm temperatures, where locations with more frequent tropical storm landfall were correlated to smaller mangroves^36^, indicating that storms can have significant impacts on mangrove productivity.

We propose that the inundation of the southern coast of Florida during some of the lowest rates of RSLR was the result of a period of high-amplitude climate variability that set off a chain of events that caused stress to the peat-accumulating mangrove forests that existed prior to the transgression that expanded Florida Bay. The timing coincides with the northern hemisphere neoglacial cooling driven by a decrease in solar insolation, which in part, drove the ITCZ southward with subsequent stabilization of ITCZ after 2.8 ka mean position southward is related to insolation-induced increases in northern high-latitude ice cover, shifting the equatorial atmospheric heat transport^68^. Despite uncertainty in regional climate predictions, observed trends in increasing temperatures and the increased frequency of abnormally wet and dry conditions in the southeastern United States over the last several decades related to the westward movement and intensification of the North Atlantic Subtropical High^52^, suggests high-amplitude shifts between wet and dry conditions, as inferred 4–2.8 ka, could significantly shorten the timeline of coastal transgression. Powerful hurricanes can damage mangrove forests and begin a complex set of responses that can lead to reduced peat accumulation, loss of mangroves, and potentially overstepping of the coast. When hurricane impacts are coupled with changes in sea-level and other climate variables such as drought, the effect can be permanent loss of the mangrove forest and reshaping of the coastline. Factoring in the impact more severe hurricanes can have on mangrove coastlines, coupled with current rates of RSLR of 2.47–3.7 mm yr^−1^ ^34,35^, which are 2–5 times the late-Holocene rate, future coastal transgressions are likely to occur rapidly and nonlinearly. These high and accelerating rates of RLSR, coupled with artificially decreased flow from Shark River (~1/2) and Taylor Slough (~2/3 to 3/4)^55^ during the 20th century, driven by construction of water control structures and water management, could further hasten the inland movement of mangrove ecosystems into the freshwater Everglades and the transgression of existing mangrove coastlines to estuarine environments.

## Methods

### Background and site selection

Florida Bay, part of Everglades National Park, is a shallow (< 3 m), inner-shelf lagoon situated south of the Florida mainland freshwater Everglades and bordered to the south and east by the Pleistocene ridge that forms the Florida Keys. Over 70% of Florida Bay is covered in mudbanks^4^, some of which are topped with mangrove-ringed islands. Florida Bay and its islands have been categorized in different ways by past researchers^4,8,9^. For this study, we focused on the central region of Florida Bay (as hydrodynamically defined^69^), which generally corresponds to the migrational zone defined by previous authors^4^, and on emergent islands that have central open mudflats, ringed by mangroves. Following Hurricane Wilma in 2005, field observations of storm deposition on the open mudflat interior of Buttonwood #7 caused us to focus on these types of islands. By selecting islands with a similar configuration, ecology, and hydrologic regime, we can make direct comparisons between the sites and interpret our findings in terms of sequencing of events, not variations in island type, plant composition, or hydrologic influence. The islands lie along a generally east–west transect of 14.8 km across the Central Zone and the north–south offset is ~4 km (Fig. 1).

### Field methods

Sediment cores were collected from the four islands in April 2014 with a Russian-style peat corer to minimize compaction. At all coring locations, we reached bedrock and obtained bedrock depth relative to the surface of the sediment (Table 1). The 10-cm long point on the Russian peat corer prevents collection of the sediment immediately overlying the bedrock, so for some cores, this material was collected with a push core, for which we could not directly account for compaction. All cores were taken inside of the mangrove berm that separates the island from the bay. Elevation measurements were made at each core location using a differential global positioning system (DGPS) with an Ashtech differential GPS (DGPS) receiver and geodetic antenna with a minimum occupation time of 30 min per site. A similar equipment combination was used to concurrently collect DGPS data at a temporary control location (base station). The base station data were post processed through the National Geodetic Survey Online Positioning User Service^70^. The rover data were post processed to the concurrent base station data in NovAtel’s GrafNav software. NOAA’s VDatum software^71^ was used to transform the site location to the North American Datum of 1983 (NAD83) reference frame and the North American Vertical Datum of 1988 (NAVD88) GEOID12A orthometric elevation, with an accuracy of ~5–10 cm. Differential geospatial positioning system (DGPS) analysis placed all coring locations below 2014 sea level (Table 1).

### Laboratory methods

Cores were transported to Reston, VA where their lithology was described, and cores were subsampled at 1-cm increments. Each core was sampled for bulk density and loss-on-ignition at 550 °C using standard procedures^72^. Bulk samples were selected for radiocarbon dating, which was performed at Beta Analytic in Miami, FL, where bulk material was acidified and picked free of roots. Age models were generated for each core using the Bayesian age-depth modeling software Bacon 2.2^73^, which applies the IntCal13 calibration curve^74^. We used median ages and calculated sediment accumulation rates using the difference in sample interval (1 cm) over the difference in median age for the given interval (Supplementary Table 1; Supplementary Fig. 1).

### Stable isotope methods

Selected samples from each core were analyzed for stable carbon and nitrogen isotopes by UC Davis Stable Isotope Facility (Supplementary Data 2). For each of those samples, bulk sediments were acidified using ~10% HCl for 24 h to remove inorganic carbon before rinsing with deionized water; this process was repeated approximately three times to ensure removal of any carbonates. Samples were dried in an oven at 40 °C, before being ground and homogenized using a mortar and pestle, then filled and weighed into tin capsules in Reston, VA. Packed sediment samples were sent to UC Davis Stable Isotope facility and analyzed for ^13^C and ^15^N isotopes using an Elementar Vario EL Cube or Micro Cube elemental analyzer (Elementar Analysensysteme GmbH, Hanau, Germany) interfaced to a PDZ Europa 20–20 isotope ratio mass spectrometer (Sercon Ltd., Cheshire, UK). Samples were combusted at 1080 °C in a reactor packed with copper oxide and tungsten (VI) oxide. Following combustion, oxides are removed in a reduction reactor (reduced copper at 650 °C). The helium carrier then flows through a water trap (magnesium perchlorate). N_2_ and CO_2_ are separated using a molecular-sieve adsorption trap before entering the isotope ratio mass spectrometer (IRMS). Final delta values are derived relative to the international standard Vienna PeeDee Belemnite (VPDB).

The isotopic signatures reflect the difference in organic source material; freshwater wetland plants and mangroves operate under C_3_ photosynthetic metabolism and have carbon isotopic values ranging from −35 to −20‰ with a typical average of −28‰ for terrestrial plants, including mangrove trees. Sea grasses, such as *Thalassia*, use a C_4_-type photosynthesis, and have an isotopic range of −18 to −6‰^20,21,75^. The transition from the more depleted range (−35 to −20‰) to the less depleted range (> −18‰) was used to indicate transition of mangrove swamps to estuarine environment.

### Pollen methods

Palynomorphs (pollen and spores) (Supplementary Data 2, Supplementary Fig. 2) were isolated using standard preparation techniques^22,76^. For each sample, one tablet of *Lycopodium* spores (batch no. 938934, x = 10,679) was added to between 0.5 and 1.5 g of dry sediment to calculate palynomorph concentration (grains/g). Samples were washed with HCl and HF to remove carbonates and silicates, respectively, and acetolyzed (one part sulfuric acid: nine parts acetic anhydride) in a boiling bath for 10 min. After neutralization, samples were treated with a 10% KOH for 10 min at 70 °C and then neutralized. Residual sample material was sieved at 149 µm and 5 µm to remove the coarse sand and clay fractions, respectively. When necessary, samples were swirled on a watch glass to remove any additional mineral material. Samples were then stained with Bismarck Brown and mounted onto slides in glycerin jelly. In general, at least 300 pollen grains and spores were counted from each sample to determine the percent abundance and concentration of palynomorphs, which can generally only be identified to genus or family level. Pollen concentrations were determined by the following formula:

1 $$Ct = ((Tc/Lc) \ast Ls)/Wts$$

Where Tc = the number of pollen taxa, Lc = number of *Lycopodium*, Ls = number of *Lycopodium* grains added to the full sample, Wts = weight of the sample^77^. We used the modern analog technique (MAT)^78,79^ to identify analogs for downcore samples from a surface sample data set of 229 samples collected throughout the greater Everglades ecosystem^22^. Dissimilarity coefficients (squared chord distance) were calculated between modern and fossil assemblages, and samples with dissimilarity coefficients ≤ 0.15 were considered to represent similar vegetational composition and, by extension, similar environmental conditions. Sample intervals were quantitatively grouped into zones using CONISS^80^. For the figures, species were grouped based on their habitat. Regional pollen is composed of pine (*Pinus)* and oak (*Quercus)*, which produce abundant pollen and are found abundantly even in marine environments. The absence of more localized, lower pollen producing taxa, such as common wetland plants, are absent from these assemblages. Freshwater marsh sums include Poaceae, Cyperaceae, *Nymphaea, Typha, Rhychus, Cladium, Schoenoplectus, Sagittaria, Crinum*, and Amaryllidaceae. Terrestrial sum includes all taxa, except the regional taxa and mangrove taxa, as well as *Batis maritima* and Amaranthaceae. Separately, presence/absence of algal cysts, dinoflagellates, and microforaminifera, or the chitinous lining of foraminifera, were noted for two cores (BA and RUS) and shown in Figs 2 and  3. Presence/absence of calcareous foraminifera and ostracode tests were combined with the presence/absence tallies from the mollusk analysis.

### Mollusk methods

Mollusks and other calcareous and invertebrate remains were examined by carefully picking apart the sediment matrix under a microscope. Samples were generally not washed or sieved in order to preserve material for other analyses, therefore, invertebrate data can only be presented as presence/absence. Mollusk specimens were identified to species level when possible, and notations were made regarding preservation and if taxa seemed abundant or common in a particular sample (Supplementary Data 3). Because the focus of this paper was the inundation of the coastal wetlands to form Florida Bay, only the lower portions of the cores were examined in detail for mollusks. Taxa were categorized by position within the coastal ecosystem (for example, freshwater, nearshore, inner estuary) based on our modern analog data set that consists of observations of 205 living species from 217 sites in Florida Bay, Biscayne Bay, and the southwest Florida tidal channels over the last 24 years^81,82^. Information from all taxa within each of the twenty categories is summarized and shown on Supplementary Fig. 3. If a single taxon was present within a given category, the category was considered present within that sample interval; if a taxon was common or abundant or if there were multiple taxa within a single category, then the category was considered common or abundant within that sample interval and indicated with an open red square.

## Supplementary information

Supplementary Information

Description of Additional Supplementary Files

Supplementary Data 1

Supplementary Data 2

Supplementary Data 3

## Data Availability

All data sets are provided in the Supplementary Information and as separate data files. Selected proxies are published online on the NOAA National Centers for Environmental Information (NCEI) Paleoclimate database.
