# Supplementary material for: Rapid inundation of southern Florida coastline despite low relative sea-level rise rates during the late-Holocene
Source: Nat Commun. 2019 Jul 19;10:3231. doi: 10.1038/s41467-019-11138-4 (PMC6642092; doi:10.1038/s41467-019-11138-4)
Supplement: Supplementary file 1 — Supplementary Information [file 41467_2019_11138_MOESM1_ESM.pdf]

Supplementary Information

Rapid inundation of the southern Florida coastline despite low relative sea-level rise rates during the late-Holocene

Miriam C. Jones *et al.*

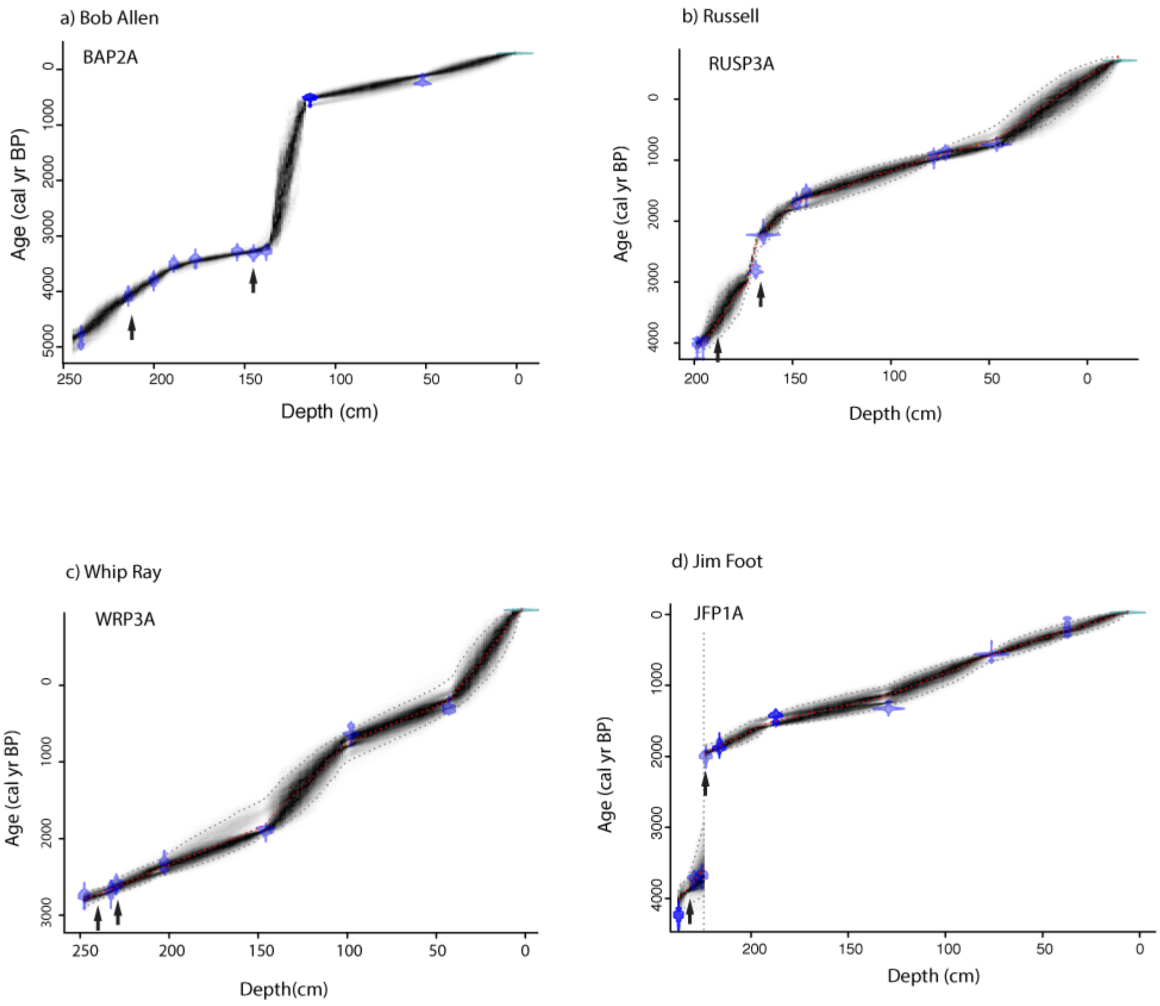

SF 1
Supplementary Figure 1
Legend: Age models for each of the four cores analyzed, generated through Bayesian age-depth modeling using Bacon[31] for a) Bob Allen, b) Russell, c.) Whip Ray (Buttonwood #7), d.) Jim Foot. Arrows indicate FMT and MET.

a) Bob Allen
(BAP-2A)

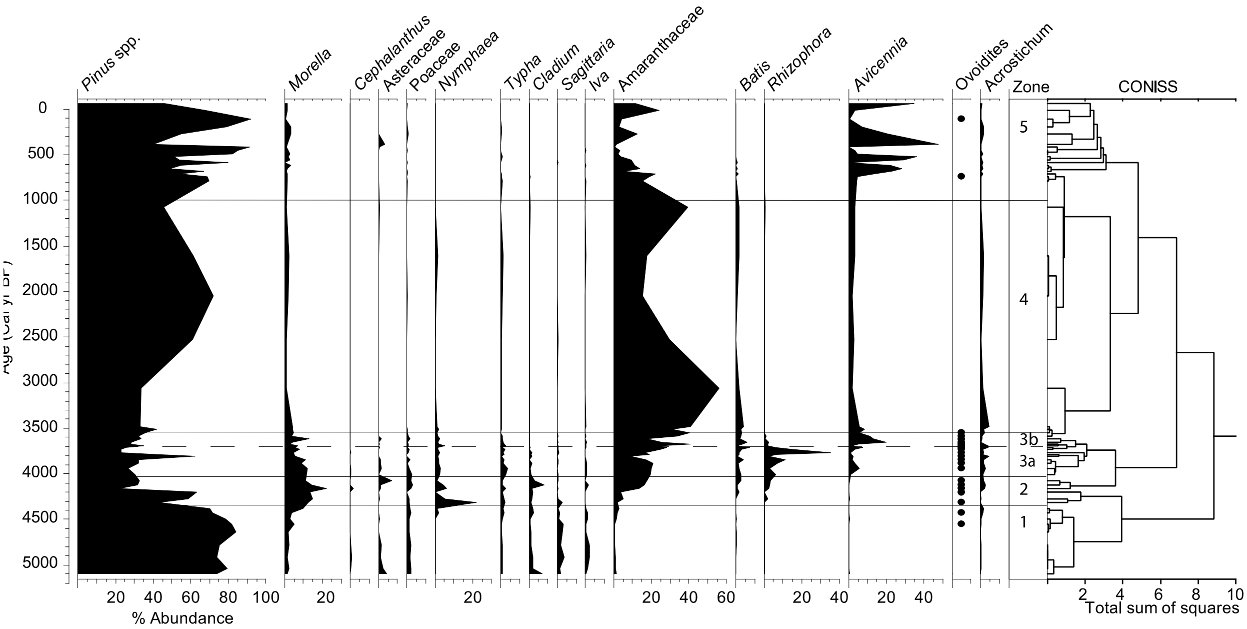

b) Russell
RUSP-3A

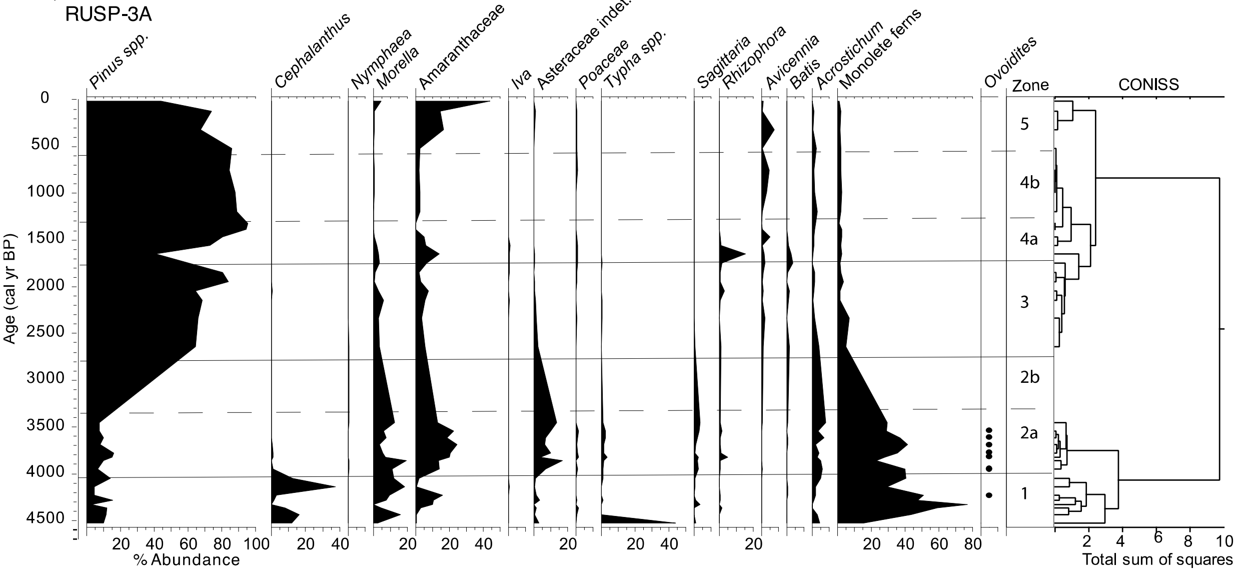

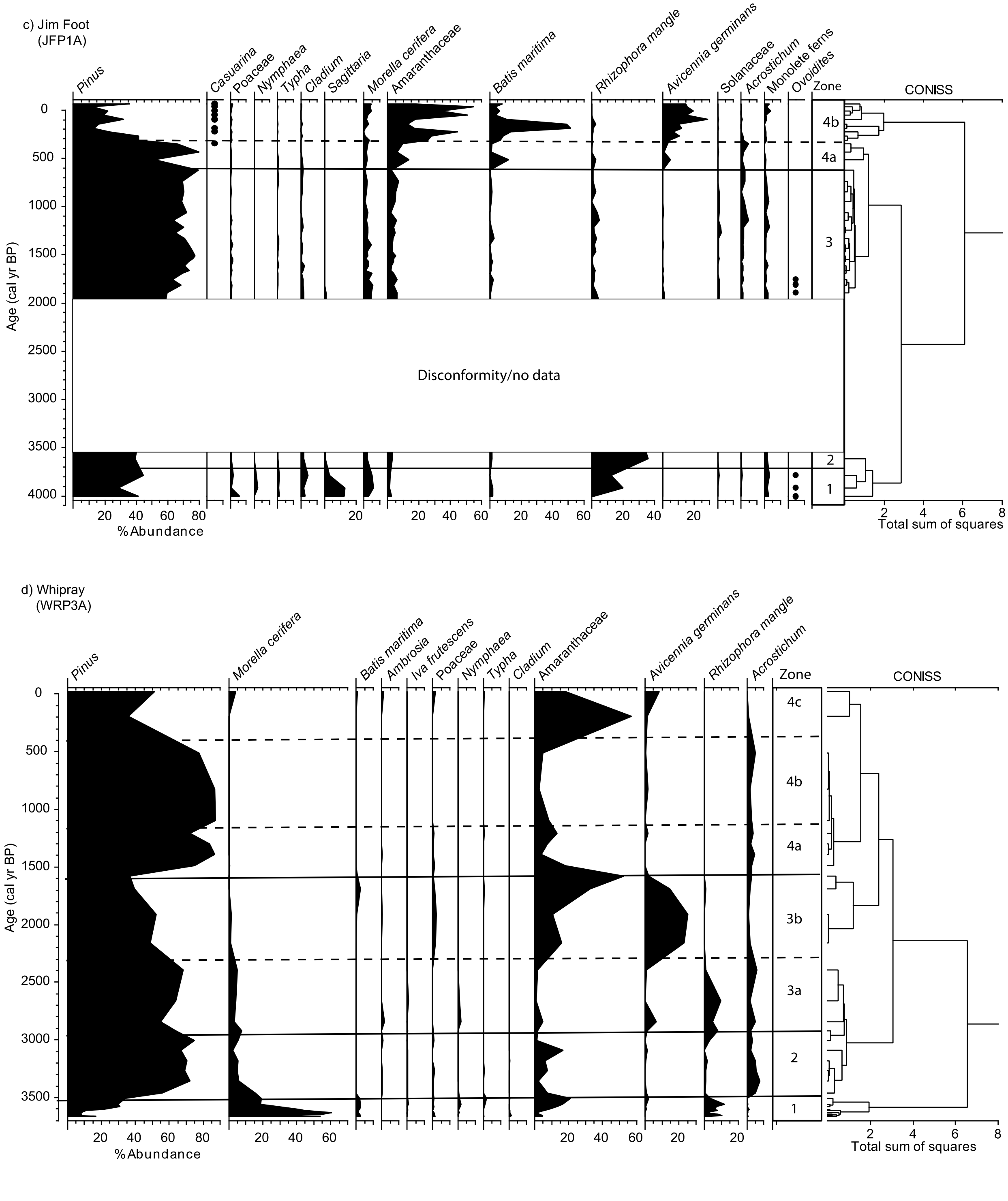

SF. 2
Supplementary Figure 2

Legend: Pollen diagrams for a) Bob Allen (BAP-2A), b) Russell (RUSP-3A), c) Jim Foot (JFP-1A), d) Whip Ray (Buttonwood #7; WRP-3A), plotted on the age scale, showing zones used in the summary figures, as determined by CONISS[39] . Taxa are shown as percent abundance, except in the case of closed circles, which indicate presence.

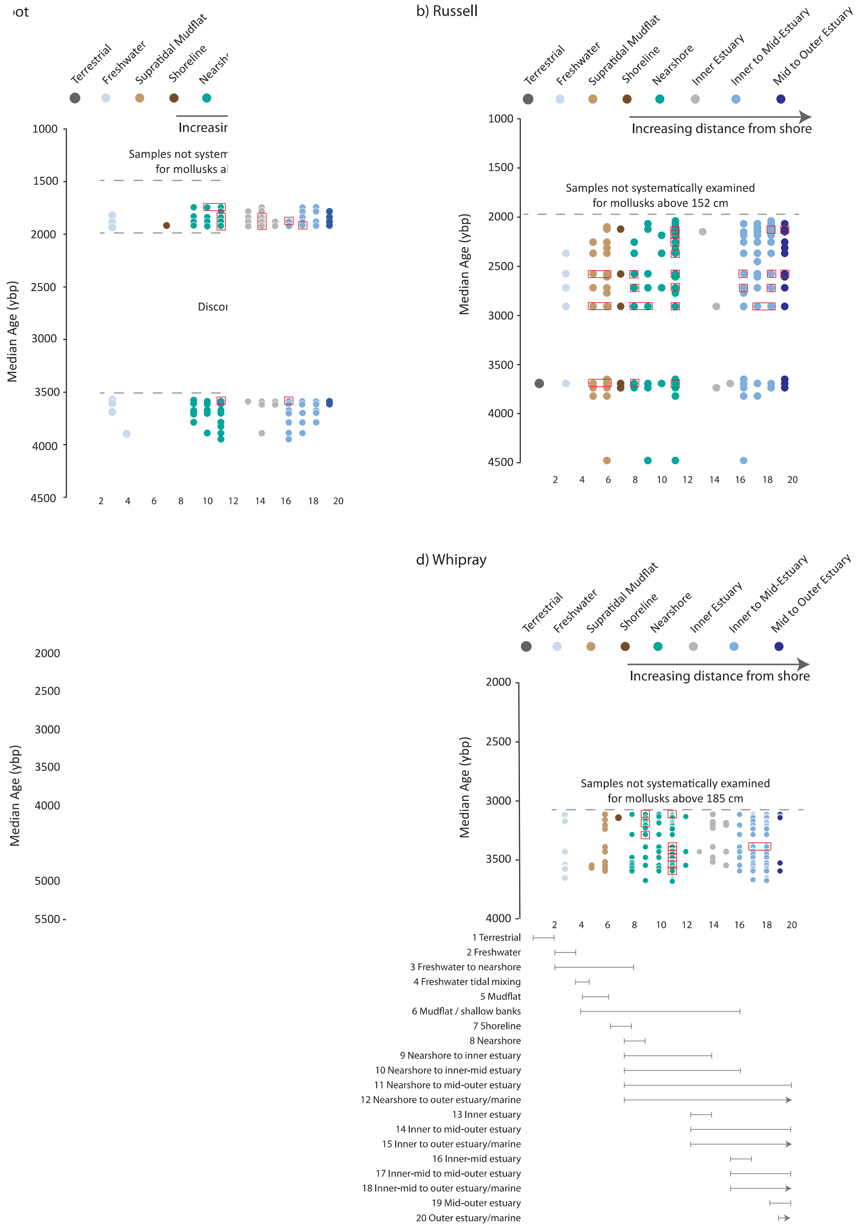

SF 3
Supplementary Figure 3
Legend: Environments indicated by mollusks (and other invertebrate and/or calcareous taxa) for lower portion of each core (a) Jim Foot (JFP-1A), (b) Russell (RUSP-3A), (c) Bob Allen (BAP-

2A), Whipray (WRP-3A), summarized from presence of individual taxa (SI Table 4). Taxa placed into 20 categories, shown at the bottom of each column, which takes into account the range of environments within the coastal ecosystem where the living taxa have been found in present-day south Florida. Red boxes indicate abundant specimens within a sample for that category.

Supplementary Table 1: Radiocarbon dates on samples.

Supplementary Table 1:
Radiocarbon dates

**Radiocarbon lab**	**Laboratory ID**	**Core ID**	**Depth (cm)**	**14C Age**	**error**
Beta	443786	BAP2A	52-53	400	30
Beta	420763	BAP2A	114-115	840	30
Beta	443787	BAP2A	145-146	3330	30
Beta	471742	BAP2A	154-155	3290	30
Beta	471743	BAP2A	177-178	3410	30
Beta	470815	BAP2A	189-190	3480	30
Beta	420764	BAP2A	200-201	3700	30
Beta	420765	BAP2A	214-215	3890	30
Beta	474604	BAP2A	240-242 - ORGANICS	4450	30
Beta	*474604*	*BAP2A*	*240-242 - PLANT (root)*	*3440*	*30*
Beta	420766	BAP2A	244-245	3790	30
Beta	434683	JFP1A	32-33	150	30
Beta	434684	JFP1A	71-72	550	30
Beta	443784	JFP1A	124-125	1360	30
Beta	443785	JFP1A	182-183	1510	30
Beta	470816	JFP1A	211-212	1890	30
Beta	434685	JFP1A	220-221	3400	30
Beta	471745	JFP1A	224-225	3430	30
Beta	434686	JFP1A	232-233.5	3790	30
Beta	447287	RUSP3A	64-65	1390	30
Beta	403580	RUSP3A	90-91	1520	30
Beta	403581	RUSP3A	96-97	1610	30
Beta	443783	RUSP3A	161-162	2100	30
Beta	470812	RUSP3A	166-167	2220	30
Beta	470812	RUSP3A	183-184	2640	30
Beta	403582	RUSP3A	187-188	3130	30
Beta	470812	RUSP3A	214-215	4030	30
Beta	403583	RUSP3A	217-218	4060	30
Beta	447288	WRP1A	47-48	760	30

Beta	387180	WRP1A	72-73	870	30
Beta	443789	WRP1A	139-140	2060	30
Beta	387182	WRP1A	212-213	3070	30
Beta	471747	WRP1A	221-222	3630	30
Beta	387183	WRP1A	237-238	4320	30
Beta	447289	WRP3A	42-43	1280	30
Beta	443791	WRP3A	97-98	1640	30
Beta	443792	WRP3A	145-146	2690	30
Beta	471746	WRP3A	203-204	3030	30
Beta	470811	WRP3A	229-230	3270	30
Beta	443793	WRP3A	232-233	3350	30
Beta	443794	WRP3A	247-248	3400	30

Supplementary Table 2: Physical properties of the cores, averaged for the different environments

**Core site**	**Environment**	**water content weight (%)**	**bulk density (g/cc)**	**OM (%)**
Russell Key	Basal freshwater	75.5 ± 3.2	0.21 ± 0.02	56.4 ± 3.7
	Basal mangrove	75.1 ± 3.6	0.2 ± 0.04	57.6 ± 3.5
	estuarine	42.2 ± 5.5	0.7 ± 0.11	10.2 ± 2.4
	Upper mangrove	75.5 ± 2.7	0.23 ± 0.03	44.1 ± 5.8
	island	29.8 ± 3.5	0.95 ± 0.13	8.5 ± 2
Whipray Key	Basal freshwater	62.9 ± 7.1	0.43 ± 0.12	39.5 ± 12.6
	Basal mangrove	59.6 ± 11.8	0.45 ±0.18	29.2 ± 16.3
	estuarine	44.4 ± 5.9	0.65 ± 0.13	13.7 ± 4
	Upper mangrove	72.2 ± 5.5	0.27 ± 0.08	38.9 ± 9.5
	island	35.7 ± 10.2	0.87 ± 0.21	11.8 ± 3.9
Jim Foot Key	Basal freshwater	76 ± 1	0.17 ± 0.02	59 ± 3.4
	Basal mangrove	57.9 ± 5.6	0.4 ± 0.14	29.5 ± 4.5
	estuarine	33.04 ± 4.2	0.82 ± 0.14	9.15 ± 1.9
	Upper mangrove	75.5 ± 5.3	0.20 ± 0.06	47 ±14
	island	58.7 ± 11.2	0.43 ± 0.17	25.3 ± 7.9
*Bob Allen Key**	Basal freshwater marl	50.3 ± 3.8	0.45 ± 0.13	9.73 ± 2.6
	Basal freshwater peat	76.9 ± 3.3	0.36 ± 0.1	68.23 ± 8.3
	Basal mangrove (red)	50.4 ± 9.1	0.73 ± 0.24	28.07 ± 8.3
	Basal mangrove (black)	34 ± 6.1	1.11 ±.26	
	estuarine	N/A	N/A	N/A
	Upper mangrove	N/A	N/A	N/A

	island	26.6 ± 3.2	1.16 ± 0.35	9.78 ± 2.5
*Biscayne Bay***	extant mangroves	83 (70-89)	0.15 ± 0.33	63-85

* cores were dried prior to bulk density measurements, so values are multipled by the water content on the dry volume

**values from reference 18

Supplementary Table 3: Regional mangrove accretion rates

**Location**	**Site name**	**Estimated age***	**Rate (mm yr^{-1})**	**Dating source**	**Reference**
Florida					
	Rookery Bay	60	1.8		19
		60	1.8	Cs-137	19
		60	2	Cs-137	19
		60	1.8	Cs-137	19
		100	1.3	Pb-210	19
		100	1.4	Pb-210	19
		100	1.6	Pb-210	19
		100	1.7	Pb-210	19
	Hutchinson Island	60	11.9	Cs-137	17
		60	6.1	Cs-137	17
		60	5.8	Cs-137	17
		60	13.3	Cs-137	17
		60	7.2	Cs-137	17
	Ten Thousand Islands	1000	1.5	C-14; not specified	17
		1000	0.7	C-14; not specified	17
	Cape Sable	1000	0.5	C-14; not specified	17
		1000	0.4	C-14; not specified	17
		1000	1	C-14; not specified	17

Hutchinson Island	1000	1.3	C-14; not specified	17
	1000	1	C-14; not specified	17
	1000	0.8	C-14; not specified	17
	1000	0.9	C-14; not specified	17
	1000	1.2	C-14; not specified	17
	1000	1.3	C-14; not specified	17
Rookery Bay Basin-1	3	2	SET, feldspar (>3yrs)	21
Rookery Bay Basin-3	3	7.55	SET, feldspar (>3yrs)	21
Rookery Bay Fringe-3	3	5.75	SET, feldspar (>3yrs)	21
	3			
Biscayne Bay (SESE)	100	4.1	Pb-210 (dating method not specified)	22
Florida Keys	60	4	Cs-137	20
	60	2.7	Cs-137	20
Rookery Bay	3	1.4	SET, Elevation table (<3 yrs)	16
Rookery Bay	3	3.7	SET, Elevation table (<3 yrs)	16
Rookery Bay	3	1.55	SET, Elevation table (<3 yrs)	16
Rookery Bay	3	7.2	Marker horizon	16
Rookery Bay	3	6	Marker horizon	16
Rookery Bay	3	5.35	Marker horizon	16
Shark River estuary (SRM)	1060	1.18	C-14	23
Shark River estuary (SRS-6)	990	1.59	C-14	23
Shark River estuary (SRS-	1200	0.81	C-14	23

	5)				
	Shark River estuary (SRS-4)	990	0.98	C-14	23
	Central FL Bay (JFP1A)	3690	0.63	C-14	(this study)
	Central FL Bay (WRP3A)	3275	1.10	C-14	(this study)
	Central FL Bay (RUSP3A)	3400	0.20	C-14	(this study)
	Central FL Bay (BAP2A)	3770	1.40	C-14	(this study)
Greater Caribbean					
	Grand Caymen	2116	0.87	C-14	24
	Grand Caymen	758	0.62	C-14	24
	Grand Caymen	842	2.48	C-14	24
	Grand Caymen	2161	0.65	C-14	24
	Grand Caymen	1319	0.86	C-14	24
	Grand Caymen	593	0.84	C-14	24
	Grand Caymen	1450	0.80	C-14	24
	Grand Caymen	1789	0.64	C-14	24
		799	1.63		
	Bermuda	3000	0.74	C-14	25
	Bermuda	4500	0.76	C-14	25
	Bermuda	2500	1.30	C-14	25
	Bermuda	1500	0.90	C-14	25
	Bermuda	500	1.30	C-14	25
	Bermuda	250	1.66	C-14	25
	Belize (Twin Cays)	230	1.96	C-14	26
	Belize (Twin Cays)	3014	0.38	C-14	26
	Belize (Twin Cays)	3744	1.04	C-14	26
	Belize (Twin Cays)	5920	0.32	C-14	26

Belize (Twin Cays)	6900	1.33	C-14	26
Belize (Twin Cays)	7240	1.18	C-14	26
Belize (Twin Cays)	7500	5.96	C-14	26
Belize (Twin Cays)	7860	2.56	C-14	26

*Pb-210 was assigned an age of 100 years, Cs-137 was assigned an age of 60 years, SETs and marker horizons were assigned an age of 3 years, Radiocarbon ages were estimated based on data provided in the literature or the midpoint between two dated intervals used to calculate the rate of accretion

Supplementary Notes 1

Results of Multiproxy Analyses

Age models for each core can be found in Supplementary Figure 1a-d. Each core was divided into zones that at first order were determined from the pollen data using CONISS[1] (Supplementary Figure 2a-d). We further refined these zones by taking into account changes in the lithology, δ^{13}C, and molluscan assemblage data. Summary figures including the lithology, stable isotopes, select pollen taxa, and mollusks can be found in Fig. 3 of the manuscript and Supplementary Fig. 4. Complete pollen data can be found in Supplementary Fig. 2a-d and Supplementary Table 3, while complete environmental information for mollusks can be found in Supplementary Fig. 3a-d and complete taxa can be found in Supplementary Table 4.

Bob Allen Key - Core BAP-2A:

Zone 1 (244 - 215 cm: 5200-4355 cal yr BP) consists of calcitic marl. *Pinus* dominates pollen assemblages (~80%), and Asteraceae, Poaceae, *Cladium,* and *Sagittaria* are present throughout (Supplementary Figure 2a). Modern analogs from the zone include freshwater marshes, specifically sawgrass marshes and wet prairies of the southern Everglades. The upper part of this zone contains the freshwater algae, *Ovoidites*, a zygospore or aplanospore of the freshwater alga *Spirogyra* (Zygnemataceae)[2,3]. *Spirogyra* is particularly abundant in shallow, stagnant, and well-oxygenated waters of freshwater marshes, and *Ovoidites* has been reported previously from freshwater peats underlying Florida Bay[4]. Freshwater marsh mollusks, Physidae and *Planorbella*, are present throughout the zone; in the upper portion, beginning ~4865 cal yr BP, hydrobiids are present, indicating a possible increase in movement of fresh water. The δ^{13}C values are < -25‰ in the C_3 photosynthetic metabolism range. Collectively, these data indicate presence of a freshwater marsh with a moderate hydroperiod.

Zone 2 (215-201 cm: 4355-4050 cal yr BP) shows a transition from calicitic marl to peat. *Pinus* pollen is less abundant (20-60%), and *Morella, Nymphaea, Typha,* and *Cladium* are more abundant. Modern analogs include sawgrass marshes and sloughs with longer hydroperiods and deeper water. The presence of *Ovoidites* throughout the zone is consistent with freshwater

conditions. Foraminifera are consistently present in this zone, indicating occasional occurrences of storm surges and/or proximity to the tidal zone. Only a few worn nearshore estuarine mollusks are found in this zone, possibly because the acidic peats prevented preservation of the relatively thin-shelled freshwater mollusks (Supplementary Figure 3c). Although δ^{13}C values increase to >-25‰ near the top of this interval, they still are well within the range of C_3 plants. Although the flora and lithology of zone 2 are typical of the relatively wetter Everglades marshes, the shift in δ^{13}C and the occurrence of a few estuarine mollusks suggests inland migration of the shoreline.

Zone 3a (201-181 cm: 4050-3730 cal yr BP) consists of peaty organic muds with greater abundance of shells. Pollen assemblages are dominated by *Pinus*, Amaranthaceae, and *Morella*, and *Rhizophora* comprises up to 40% of assemblages. *Avicennia* and *Batis* are common (up to 5%) of assemblages. Modern analogs for these assemblages consist primarily of mangroves, indicating proximity to the shoreline and saline influence; however, the presence of *Ovoidites* and other freshwater taxa (*Nymphaea, Typha, Cladium*) suggests the site still was influenced by freshwater flow. The δ^{13}C remains in the C_3 photosynthetic range of -24 to -23‰. Mollusks typical of mudflat and nearshore environments dominate assemblages, and hydrobiid presence indicates freshwater flow; however, the assemblage includes the full range from terrestrial gastropods to a few mid-outer estuarine taxa, which likely were deposited during storm surges. Larger freshwater marsh molluscan taxa are absent. Combined with the presence of common foraminifera and ostracods, it appears that the site shifted from a predominantly freshwater marsh to a nearshore environment with both estuarine and freshwater influx.

Zone 3b (181-145 cm: 3730-3520 cal yr BP) includes a transition from peat to carbonate mud at ~3600 cal yr BP. Pollen assemblages shift from dominance of *Rhizophora* to *Avicennia*, Amaranthaceae generally increases, and *Batis maritima* remains present. Freshwater taxa (*Nymphaea*, *Typha, Ovoidites*) remain present in small numbers, although *Cladium* decreases in this zone. Modern analogs for samples deposited before 3600 cal yr BP include southern Everglades sawgrass marshes and wet prairies, but analogs for the remainder of the zone are from Florida Bay playas. The δ^{13}C decreases from ~-23‰ at the zone boundary to ~-25‰ for the remainder of this zone. The predominant molluscan taxa in this interval are species typically found on interior island mud flats or shallow mudbanks in present day Florida Bay (*Polymesoda floridana*, *Cerithidea scaliformis*, *Cerithidea costata*). Hydrobiids are present in some intervals, indicating occasional freshwater influx. Scattered shells of nearshore and inner to outer estuarine shells are present, implying transport by storm surges. Foraminifera and ostracodes are relatively common in this zone. Collectively, the proxies in zone 3b suggest a cessation in the encroachment of the estuary and establishment of the island. Island establishment indicates that the rate of sediment accretion outpaced sea level rise at the time.

Zone 4 (145-119 cm: 3520-1110 cal yr BP) consists of carbonate mud with some organics and no visible shells. Amaranthaceae and *Pinus* dominate pollen assemblages, with Amaranthaceae exhibiting peak abundances at ~3200 cal yr BP (55%) and ~1100 cal yr BP (40%). *Avicennia* pollen remains present in low percentages (<5%). Dinoflagellates *Polysphaeridium zoharyi* and *Spiniferites* are present throughout this zone, suggesting higher salinity water reaching the site, but the intermittent presence of *Ovoidites* indicates the site also received freshwater. Although δ^{13}C values are slightly less depleted (-21‰) than in previous zones, they are not indicative of C_4-dominance, as was seen in estuarine phases of other cores. Although molluscan remains were not examined systematically in samples younger than ~3400

cal yr BP, those observed indicate continued dominance of the mud-flat and/or shallow mudbank species that dominated zone 3b. Hydrobiids were present in some intervals, and nearshore to outer estuarine taxa were scattered throughout the interval l (Supplementary Figure 3c). Proxies in zone 4 are consistent with deposition on a mudflat, either on an island or behind a coastal berm with pools that occasionally filled with either rain water or estuarine water.

Zone 5 (119-0 cm: 1100 cal yr BP to present) consists of carbonate muds with scattered organics and no visible shells Pollen assemblages are dominated by *Pinus* and *Avicennia* (up to 45%), with lower abundance of Amaranthaceae. Modern analogs for pollen assemblages are playa sites and wet prairies in Taylor Slough. $\delta^{13}C$ was variable, ranging from -24‰ to nearly -15‰. Freshwater algae (*Ovoidites)* foraminifera, and dinocysts (*P. zoharyi* and *Spiniferites)* are present, indicating both freshwater and estuarine influx to the site. Mollusks were not examined in this segment. The increase in *Avicennia* pollen suggests that this zone marks either the expansion of the island itself, or an increase in the vegetation across the island. The lithology and pollen are consistent with what is seen on the island today; shifts in the $\delta^{13}C$ to C_4-like photosynthesis are likely due to deposition of seagrasses during storm events.

Russell Key - Core RUSP-3A:

Zone 1 (217-208 cm: 4600-4185 cal yr BP) is composed of dark peat with $\delta^{13}C$ values between -29 to -26‰. The lowermost sample of this core (~4600 cal yr BP) is dominated by *Typha* (45%), with common *Cephalanthus* (15%), *Acrostichum* (5%), and *Pinus* (10%) (Supplementary Figure 2b). Although there were no modern analogs for this sample, the combined dominance of *Typha* and presence of *Nymphaea, Cladium,* and *Sagittaria* are indicative of a freshwater marsh environment. Assemblages in the rest of the zone (~4500 to ~4000 cal yr BP) are dominated by monolete fern spores (~80%) and common occurrence of *Cephalanthus, Morella,* and Amaranthaceae. Modern analogs for these samples are Everglades tree islands, which are characterized by dominance of fern spores[5], suggesting generally drier conditions and the build-up of a tree island. Only a few scattered, worn mollusks were visible in this zone, ranging from mudflat/nearshore species (*Anomalocardia cuneimeris*) to inner-mid outer estuarine species (*Parastarte triquetra*). No freshwater mollusk species were found. The flora, dark peat, general lack of invertebrates, and the isotopic ratios indicate a freshwater marsh shifting to a tree island in zone 1; however, the occurrence of a few scattered estuarine mollusks suggests proximity to the coast (Supplementary Figure 3c).

Zone 2a (208-190 cm: 4185-3320 cal yr BP) consists of dark peat, similar to zone 1, with pockets and/or layers of shell hash in the middle of the zone (~3820 - 3690 cal yr BP). Monolete fern spores dominate the assemblages (25-40%), and Amaranthaceae and Asteraceae pollen are subdominant. *Typha, Sagittaria,* and Cladium, and *Ovoidites* are present throughout the zone, and *Rhizophora* pollen is present in most samples. Modern analogs include sawgrass marshes and sloughs in the central and southern Everglades and mangrove forests in southwest Florida. The $\delta^{13}C$ ranges from -27 to -25‰. Mollusks in the shell layer include from terrestrial, nearshore, and mid-outer estuarine taxa. The predominant mollusk species are typical of mudflats (*Polymesoda floridana*), mudflats or nearshore shallow estuarine zones (*Anomalocardia cuneimeris, Cerithidea costata*), and nearshore estuarine (*Acteocina canaliculata*). Foraminifera and ostracodes are also abundant in the shell hash. The dark peat,

pollen assemblages, and isotopic ratios indicate that freshwater marshes occupied the site, with mangroves nearby; the presence of shell hash in the zone confirms proximity to the shoreline and deposition of shells during a period of increased storm overwash.

Zone 2b (190-185 cm: 3320-2745 cal yr BP) includes an abrupt transition from peat to a shell layer overlain by a shelly, organic-rich, carbonate mud. No pollen samples were prepared from this zone. The δ^{13}C remains ~-25‰ in this zone. A diverse mollusk assemblage preserved in the shelly layer includes taxa from environments ranging from mudflats to the mid-outer estuary. Taxa present include mudflat species (*Polymesoda floridana*), those from mudflats or nearshore shallow estuarine zones (*Anomalocardia cuneimeris*, *Cerithidea costata*), and those from nearshore estuaries (*Acteocina canaliculata*). Also common are nearshore to inner estuary taxa (the gastropod *Bittiolum varium* and barnacles) and nearshore to mid-outer estuary taxa (*Brachidontes exustus*, *Cerithium muscarum*). The lithology, isotopic ratios and mixed molluscan assemblage indicate deposition, perhaps during storm surges, near the shoreline.

Zone 3 (185-127 cm: 2745-1800 cal yr BP) consists of a basal shell-rich carbonate mud mixed with peat fragments that grades upward into carbonate mud with some organics and fewer shells. Pollen assemblages are dominated by *Pinus* (60-80%) with low percentages of *Morella, Typha, Rhizophora, Avicennia,* and monolete ferns. Modern analogs include southern Everglades sawgrass marshes and wet prairies. Foraminifera and dinoflagellates (*Polspaheridium zoharyi* and *Spiniferites)* are present throughout this zone, and *Ovoidites* is present in three samples. Although δ^{13}C or the lowest sample was -25‰, they abruptly transitioned to -18‰, reaching -15‰ by the top of the zone. These values indicate a shift to C_4 type plants, such as sea grasses. Although mollusks were not systematically examined above ~2050 cal yr BP, species present in the lower portion of the zone indicate a progression away from the shoreline into deeper water. From ~2760 to ~2368 cal yr BP, hydrobiids are present, indicating proximity to shore and a freshwater influx. Species indicative of mudflats and nearshore environments (*Polymesoda floridana*, *Cerithidea scaliformis*, *Cerithidea costata*, *Melampus* spp.) are present from ~2760 to 2255 cal yr BP. The most common mollusk species throughout the examined portion of zone 3 (~2760 to 2050 cal yr BP) are those that tolerate a nearshore to outer estuarine conditions (*Brachidontes exustus*, *Bulla striata*, *Cerithium muscarum*). Collectively, the lithology, pollen, isotopic ratios, and molluscan assemblage indicate a shift to estuarine conditions, likely near an outflow of the freshwater environment, given the presence of freshwater mollusks and some freshwater marsh pollen.

Zone 4a (127-86 cm: 1800-1435 cal yr BP) consists of a basal carbonate mud with increasing organic content toward the top of the zone. Pollen assemblages of the basal sample (~1700 cal yr BP) are characterized by peak abundance of *Rhizophora* (20%) and Amaranthaceae and decreased *Pinus* (80%). Modern analogs for this assemblage include dwarf mangroves of Taylor Slough, other mudbank/playa sites, and southern Everglades wet prairies. The dinocysts *P. zoharyi* and *Spiniferites* also are present in the basal sample. Pollen assemblages from the rest of the zone (1647 - 1435 cal yr BP) are dominated by *Pinus*, with *Avicennia*, Amaranthaceae, and Poaceae present. Modern analogs for these samples include southern Everglades sawgrass marshes and wet prairies. Foraminifera are consistently present in this zone, except for the top sample. The δ^{13}C values range from ~-15 to -20‰, consistent with *Rhizophora* peat accumulation. Mollusks were not systematically examined in this zone, but

estuarine species (*Crepidula* spp. and *Parastarte triquetra*) were observed. The decrease in the isotopes and occurrence of *Rhizophora* and *Avicennia* pollen in this interval likely represents the emergence of the present-day island.

Zone 4b (86-33 cm: 1435-570 cal yr BP) shows a transition from an organic carbonate mud to a dense carbonate mud. Pollen assemblages are overwhelmingly dominated by *Pinus* (85-96%), and *Avicennia* is present throughout the zone. Modern analogs include wet prairies in the southern reaches of Taylor Slough. The $\delta^{13}C$ increased from ~-23‰ to ~18‰. Although foraminifera are absent, the dinocysts *P. zoharyi* and *Spiniferites* are present throughout. *Ovoidites* is present in one sample near the top of this zone. Mollusks were not examined systematically in this zone, and no mollusks were detected during sampling for other proxies. This zone represents an expansion of the island edge outward from the core site, as evidenced by the decrease in peat and increase in carbonate mud.

Zone 4c (33 - 0 cm: 570 cal yr BP to present) shows a transition from dense carbonate muds to white carbonate clay with some organics. Pollen assemblages are characterized by increasing abundance of Amaranthaceae to 40-50% and a corresponding decrease in *Pinus.* *Avicennia* is present throughout, decreasing from 10% to 5% during the last 100 years. $\delta^{13}C$ remained ~-15‰, suggesting continued influence of sea grasses, likely deposited during storm surge events. Foraminefera are present in one sample and *P. zoharyi* and *Spiniferites* are present throughout this zone. This zone represents the present-day interior island carbonate mud environment, with no vegetation growing at the core site.

Jim Foot Key: Core JFP-1A:

Zone 1 (233-227 cm: 4000-3825 cal yr BP) consists of peat with scattered shell fragments and some mottling with carbonate mud. Within the upper unit, sand-sized shell fragments occur, indicating proximity of a high-energy environment. Pollen assemblages are dominated by *Pinus*, with common occurrence of *Morella*, *Sagittaria*, *Cladium*, and Poaceae (Supplementary Figure 2c). Modern analogs for the lower sample are sawgrass marshes in the southern Everglades. *Ovoidites* spores are present in all samples. $\delta^{13}C$ values range from -26‰ to -25‰. Although mollusks are sparse and mostly worn in this interval, the species that are present represent environments ranging from nearshore to mid-outer estuary. Most common are worn fragments of *Brachidontes exustus* (Supplementary Figure 3a). Collectively, the proxies indicate initial deposition in a freshwater marsh near the coast.

Zone 2 (227-219 cm: 3825-3590 cal yr BP) consists of peat/mud mixture with shells starting at 3825 cal yr BP. Pollen assemblages are co-dominated by *Pinus* and *Rhizophora*, with decreasing freshwater taxa from zone 1. The lower two samples have no analogs in the modern dataset, and the upper sample is analogous to dwarf mangroves near Florida Bay. $\delta^{13}C$ values range from -25‰ to -24‰. Mollusks deposited between 3825 and 3590 cal yr BP generally are worn, and are indicative of nearshore to mid-outer estuary species. The most common are *Bittiolum varium*, *Brachidontes exustus*, *Bulla striata*, and *Cerithium muscarum*. Near the upper part of the zone an increasing number of species that live farther out in the estuary appear, such as *Chione elevata*, *Prunum* spp., *Modulus modulus*, and *Cerithium eburneum*. Foraminifera and

Ostracoda are also common in the upper portion of the zone. These proxies indicate a mangrove environment near the shoreline. Evidence for increasing energy and transport in the upper part of the zone, suggest that the site was close enough to the shoreline to be affected by storm surges. Based on closely spaced radiocarbon dates, no sediment record exists between ~3500 to ~2000 cal yr BP, indicating the presence of a disconformity in this part of the record.

Zone 3 (218 - 76 cm: ~2000 to 575 cal yr BP) transitions from a carbonate mud with abundant shell fragments and fine organics to a carbonate mud with no visible shells. Pollen assemblages are dominated by *Pinus* (60-80%), with common occurrence of *Morella* (<10%) and Amaranthaceae (<10%). *Rhizophora* is consistently present in this zone, but at low percentages (~5%), and *Cladium, Typha,* and Poaceae are present in the first half of this zone (<5%). Modern analogs include sawgrass marshes and wet prairies throughout the southern Everglades. $\delta^{13}C$ values range from -17 to -14‰ in this zone. Samples were examined for mollusks from ~2000 to ~1500 cal yr BP, but no mollusks were identified above ~1740 cal yr BP. The species present are very similar to zone 1. The dominant species are *Brachidontes exustus*, *Bulla striata*, and *Cerithium muscarum*; also *Pteria* sp. These species are associated with seagrass beds or algal mats in nearshore/inner estuary to mid-outer estuary habitats. Foraminifera and ostracodes are common in the lowest portion of the zone. The dominance of the regional pollen (*Pinus)* and the $\delta^{13}C$ values consistent with C_4-like seagrasses suggests that deposition during this interval is most consistent with the estuarine phase.

Zone 4a (76-50 cm: 575 cal yr BP to 345 cal yr BP) transitions from a carbonate mud with no visible shells to an organic-rich carbonate mud, with a shell hash layer ~20 cm thick from ~525-345 cal yr BP. Pollen assemblages are dominated by *Pinus* pollen, Amaranthaceae, *Batis*, and *Avicennia* abundance increased relative to zone 2. Modern analogs include both Florida Bay playas and wet prairies in Taylor Slough $\delta^{13}C$ values shifted from ~-15‰ to -22‰, indicating increasing influence from mangroves. Mollusks were not examined in this interval. The transition in the carbon isotopes and pollen assemblages suggest island establishment in this zone.

Zone 4b (50-0 cm: 345 cal yr BP to present) consists of a basal calcareous mud with abundant shell fragments and small amounts of peat and transitions to a peat from ~190 to 100 cal yr BP. The peat is overlain by a carbonate mud with evidence of reddish algae during the last 100 years. *Pinus* pollen decreased to ~10% in this zone, and *Avicennia* increased from ~10% at the base to ~25% in the uppermost sample. Amaranthaceae abundance varies from 20-40%, and *Batis* abundance peaked between ~140 and 250 cal yr BP. Florida Bay playa sites represent the only modern analogs for samples in this zone. $\delta^{13}C$ values in estuarine muds under- and overlying the peat are ~-18‰, but they decreased to -26‰ during peat accumulation. The pollen assemblage, isotopes, and lithology of the upper portion of zone 4b are consistent with the environment of the present-day island.

Whipray Key - Core WRP-3A:

Zone 1 (256-231 cm: 3700- 3550 cal yr BP) is composed of a dark peat. Pollen assemblages are dominated by *Morella* and *Pinus*, with common *Rhizophor*a, *Avicennia*, and *Batis* (Supplementary Figure 2d). Freshwater taxa, including *Nymphaea*, *Typha*, and *Cladium*, also are

present. Modern analogs for this zone include brackish marshes and dwarf mangroves. $\delta^{13}C$ values range from -26‰ to-24‰. A diverse molluscan assemblage is present. Freshwater hydrobiids and species indicative of mudflats or very nearshore environments (*Polymesoda floridana*, *Cerithidea scaliformis*, *Cerithidea costata*) are present but generally poorly preserved. The most common mollusk species in this interval are *Brachidontes exustus* and *Cerithium muscarum*, species that can range from nearshore to mid-outer estuary. *Modulus modulus*, *Prunum* sp. and other species from farther out in the estuary also are present (Supplementary Figure 3d). Foraminifera and ostracodes are relatively common in the uppermost samples of this zone. Floral, faunal, lithologic, and isotopic ratios indicate the presence of fresh- to brackish marshes near a mangrove shoreline, and the presence of mid-estuarine mollusks suggest an increase in storm surge deposition.

Zone 2 (231-171 cm: 3550-3000 cal yr BP) transitions from a firm, dark, organic-rich mud at the base to an organic-rich carbonate mud with visible shell fragments and macro-plant material near the top. Pollen assemblages are dominated by *Pinus*, with common Amaranthaceae and *Morella*. *Rhizophora* and *Acrostichum* (a fern commonly found in mangrove habitats) are common throughout the zone, and *Avicennia* is present in lower abundances. Modern analogs include dwarf mangroves near Florida Bay and other playa sites. $\delta^{13}C$ transitions maintain values of -18‰ to -16‰, indicating that the organic fraction of the sediment is dominated by C4-like seagrasses. Mollusks were systematically examined only in the lower portion of the zone, from ~3550 up to ~3130 cal yr BP. Presence of scattered hydrobiids indicates proximity to a freshwater source. Several species indicative of nearshore / mudflats are present (*Anomalocardia cuniemeris* and *Cerithidea costata*). *Brachidontes exustus*, *Cerithium muscarum*, and *Bittiolum varium* are the most common species in this interval, and they are indicative of nearshore to inner or mid-estuarine environments. Also present are *Crepidula* spp., *Modulus modulus*, *Prunum* sp., and other species that range from inner to outer estuary. A more marine influence between ~3160 to 3133 cal yr BP is indicated by the presence of *Halimeda* (a calcareous algae) and *Cerithium eburneum*. Foraminifera and ostracodes are found throughout the zone and are abundant in a few samples. The lithology, pollen assemblages, isotopes, and fauna indicate that this site was submerged but probably close to shore throughout the time of deposition.

Zone 3a (171-128 cm: 3000-2345 cal yr BP) consists of a basal organic-rich carbonate mud with visible shells that transitioned to a peaty, very organic rich mud ~2770 cal yr BP. Although *Pinus* assemblages are dominated by *Pinus*, *Rhizophora* and *Acrosticum* are common elements. Marsh taxa (Asteraceae and Nymphaea) are present in low abundances. Only the upper and lowermost samples in the zone have modern analogs, which are dwarf mangrove sites near Florida Bay. $\delta^{13}C$ values decrease from -19‰ to -24‰, likely reflecting by the increasing presence of *Rhizophora.* Mollusks were not examined in this zone. The lithology, pollen assemblage, and isotopes indicate the re-establishment of terrestrial habitats, most likely the formation of the present-day island, with freshwater environments nearby.

Zone 3b (128-100 cm: 2345-1475 cal yr BP) consists of a mottled peat and light tan carbonate mud, with organic content decreasing upward. By ~1800 cal yr BP the sediment is a light tan carbonate mud with no visible shells. Pollen assemblages are characterized by a sharp increase in *Avicennia* (>20%) and Amaranthaceae (up to 55% at the top of this zone) and

decreased abundance of *Rhizophora* (<5%). *Batis* also increases (~5%) near 1600 cal yr BP, and Poaceae and *Acrostichum* are present throughout this zone. Modern analogs are other playa sites from Florida Bay. Mollusks were not examined in this zone. The $\delta^{13}C$ remains near -25‰, indicating less influence by seagrasses. The lithology suggests formation of a mudflat and the pollen assemblage and isotope values suggest expansion of the island.

Zone 4a (100-52 cm: 1475-1215 cal yr BP) consists of a carbonate mud. Pollen assemblages are dominated strongly by *Pinus* and Amaranthaceae and *Avicennia* comprises <10% and <5% of assemblages, respectively. Modern analogs are wet prairies of the southern Everglades. $\delta^{13}C$ remain at -25‰. The pollen assemblage, lithology and isotopes indicate continued deposition on an island interior mudflat with either decreased abundance or greater distance from mangroves.

Zone 4b (52-18 cm: 1215-480 cal yr BP) consists of a relatively firm carbonate mud. Pollen assemblages are dominated strongly by *Pinus* and *Avicennia* and *Acrostichum* are present throughout. Modern analogs are wet prairie sites near Florida Bay. $\delta^{13}C$ values increase to -18‰. The environment indicated by zone 4b is similar to zone 4a, with greater abundance of *Avicennia*; the shift in isotope ratios suggests more deposition of seagrasses on the island and possibly an increase in storms.

Zone 4c (18-0 cm: 480 cal yr BP to present) is a slightly more organic-rich carbonate mud than the underlying zone 4b. Pollen assemblages are dominated by Amaranthaceae (60%), and *Morella*, *Ambrosia*, *Batis*, *Avicennia*, and Poaceae were more abundant in the last 100 years. Modern analogs include playa sites from Florida Bay. $\delta^{13}C$ remains at -18‰. Pollen assemblages, lithologic, and isotopic evidence suggest conditions similar to today throughout this interval.

Supplementary Notes 2
Paleo-environment and sea-level determination

Bedrock topography and previous analyses place three out of the four coring locations into proposed paleo-drainage channels of Taylor Slough[6] (Fig 1), which today represents a ~4-5% of total flow volume (calculated from data in 67) of the entire Everglades drainage. Analyses from those three cores, which include Russell Key, Whipray Key, and Jim Foot Key reveal that freshwater peats began accumulating on bedrock 4.5-3.7 ka, consistent with other studies[4,7,8], indicating sufficient freshwater flow for peat initiation and accumulation[9]. The fourth core, from Bob Allen Key, began as a freshwater marl-precipitating marsh at ~5.2 ka, analogous to marl marshes in portions of the Everglades today, and transitioned to a freshwater peat ~4.7 ka. High-resolution radiocarbon dates on each core provide the most detailed information available on the timing of the transition from freshwater to mangrove to estuarine environment as sea level rose in the late Holocene. Results from pollen analysis show that the transition from freshwater peat to mangrove peat occurred between 4.2-3.5 ka (Fig. 2, Supplementary Fig. 1). Previous analyses on buried mangrove peats and corals indicate this period corresponds to a slow-down in RSLR from a higher rate of 0.83 - 2.3 mm/yr[4,8] to a lower rate 0.4 – 0.7 ±0.3 mm/yr[4,8,9,10] by 3.2 ka.

For each core, we used a combination of lithology, pollen assemblages, molluscan assemblages, and stable carbon to interpret changes in depositional environment. The change from basal freshwater peat to mangrove peat was determined by an increase in *Rhizophora* pollen and a decrease in freshwater palynomorphs (e.g., *Morella cerifera, Cladium* spp., *Nymphaea*). In most cases, the shift from freshwater to mangrove assemblages was also accompanied by a 1-2‰ shift to less depleted $\delta^{13}C$. C_3 plants have a distinct isotope signature (<-22‰) compared to C_4 plants (>-18‰)[12,13,14], and seagrasses, while not strictly C_4, have a signature that resembles C_4 photosynthesis[12]. Therefore, we used the shift in carbon isotope composition of the organic carbon fraction from ~-24 to -26 ‰ to -14 to -18 ‰, as well as a decrease in the mangrove pollen taxa and increase in *Pinus*, a regional pollen type that dominates an assemblage in the absence of other taxa, as an indication of a shift toward estuarine conditions. Once that transition was determined based on the pollen assemblages, we used the carbon isotopes to determine the number of years it took for a single core to go from its lighter (more depleted) values to its heaviest (least depleted) values as the time it took for the formerly coastal environment to become fully submerged.

We used the sum of the core depths (at the freshwater-mangrove and mangrove-estuarine transitions) and the surface elevation of the core site derived by the dGPS (which all were below modern sea level, Table 1) to track late-Holocene sea level relative to modern sea level and calculated a rate of sea-level rise in Florida Bay from 4500 to 2740 cal yr BP of 0.67 mm yr^{-1}. This falls near the published range of 0.6 mm $yr^{-14,10}$ and 0.7 - 0.3 mm yr^{1} after 3900 cal yr BP, but lower than the rate of ~0.9 mm yr^{-1} (7000-2000 cal yr BP)[11]. In all cases, the transition from freshwater to mangrove was inevitable due to the low accretion rates of the freshwater peat compared to the rate of RSLR at the time of transition, even accounting for compaction. However, based on the pre-inundation accretion rates of mangrove peats (Table 1), our data show that all cores have higher apparent accretion rates than the rate of RSLR at the time and should have been able to maintain their position above sea level between ~4000 to 2700 cal yr BP, assuming that the apparent rate of accretion in the core represents the net vertical accretion – elevation change. Modern vertical accretion observed in Florida *Rhizophora mangle* fringe forests were 7.5± 0.8 mm yr^{-1}, despite an elevation change of 2.4±0.5 mm yr^{-1}, indicating shallow subsidence comprised 5.1 mm yr^{-1} over the 1-2.5-year study period[4]. Comparing slightly longer timescales, the difference between accretion on decadal (^{137}Cs and ^{210}Pb) timescales versus radiocarbon timescales, shows average short-term accretion rates of Florida mangroves of 1.8 and 1.6 mm yr^{-1} and long-term rates of 1.1 mm yr^{-1}, respectively[17], suggesting a compaction or decomposition of 35 ± 4% from ^{210}Pb to ^{14}C timescales. Comparing annual accretion to long-term accretion, results in a compaction (Supplementary Table 2) with of 85% from annual accretion rates (Supplementary Table 3; Fig. 7). We evaluated the compaction rate by comparing the bulk densities and water content of the mangrove peat sections of the studied Florida Bay cores (Supplementary Table 2) with uncompacted continuous mangrove peats from Biscayne Bay[18] and determined that the peats compacted 9-39%, based on the difference in water content between the uncompacted and compacted peats. The bulk densities for the mangrove sections in our core were significantly higher than those from the uncompacted peats, and yielded compaction rates of 25-66%. Given the lower organic matter content for the mangrove portions of the core compared to the continuous peats, we conclude that the difference in bulk density and water content is not entirely due to compaction. Therefore, we use the more conservative range for compaction of 9-30%. Given the potential for compaction, the observed (calculated) rates of

accretion from the late Holocene peats in the studied cores are minimum accretion rates and were likely much higher at the time of deposition, making it likely that all cores were accreting sediments or peat at a higher rate than the rate of sea-level rise at the time of deposition.

We plotted the latest sea-level curve for South Florida[10], which updates previous curves[11,15].

Supplementary References

1. Grimm, E.C. CONISS: a FORTRAN 77 program for stratigraphically constrained cluster analysis by the method of incremental sum of squares. *Computers & geosciences*, *13*(1), 13-35 (1987).

2. Van Geel, B. and Grenfell, H.R. Spores of Zygnemataceae, *in* Jansonius, J. and McGregor, D.C. (eds.), Palynology: principles and applications. *American Association of Stratigraphic Palynologists Foundation*, 1, 173-179 (1996).

3. Rich, F., Kuehn, D., and Davies, T.D. The paleoecological significance of *Ovoidites*. *Palynology*, 6, 19-28 (1982).

4. Scholl, D.W. Recent sedimentary record in mangrove swamps and rise in sea level over the southwestern coast of Florida: Part 2. *Marine Geology*, *2*(4), 343-364 (1964).

5. Willard, D.A., Weimer, L.M. and Riegel, W.L. Pollen assemblages as paleoenvironmental proxies in the Florida Everglades. *Review of Palaeobotany and Palynology*, *113*(4), 213-235 (2001).

6. Lidz, B.H., Reich, C.D., and Shinn, E.A. Systematic mapping of bedrock habitats along the Florida Reef Tract – Central Key Largo to Halfmoon Shoal (Gulf of Mexico). *U.S. Geological Survey Professional Paper* 1751 (2007).

7. Enos, P. and Perkins, R.D. Evolution of Florida Bay from island stratigraphy. *Geological Society of America Bulletin*, *90*(1), 59-83 (1979).

8. Wanless, H.R., Parkinson, R.W. and Tedesco, L.P., Sea level control on stability of Everglades wetlands. *Everglades: the Ecosystem and Its Restoration. St. Lucie Press, Delray Beach, FL, USA*, 199-223 (1994).

9. Willard, D.A. and Bernhardt, C.E. Impacts of past climate and sea level change on Everglades wetlands: placing a century of anthropogenic change into a late-Holocene context. *Climatic Change, 107(1-2),* 59 (2011).

10. Khan, N.S., Ashe, E., Horton, B.P., Dutton, A., Kopp, R.E., Brocard, G., Engelhart, S.E., Hill, D.F., Peltier, W.R., Vane, C.H. and Scatena, F.N. Drivers of Holocene sea-level change in the Caribbean. *Quaternary Science Reviews*, *155*, 13-36 (2017).

11. Scholl, D.W. and Stuiver, M. Recent submergence of southern Florida: a comparison with adjacent coasts and other eustatic data. *Geological Society of America Bulletin* 78, 437-454 (1967).

12. Hu, X., Burdige, D.J. and Zimmerman, R.C. δ13C is a signature of light availability and photosynthesis in seagrass. *Limnology and Oceanography*, *57*(2), 441-448 (2012).

13. Khan, N.S., Vane, C.H. and Horton, B.P. Stable carbon isotope and C/N geochemistry of coastal wetland sediments as a sea level indicator. *Handbook of Sea-Level Research*, 295-311 (2015).

14. Hemminga, M.A. and Mateo, M.A. Stable carbon isotopes in seagrasses: variability in ratios and use in ecological studies. *Marine Ecology Progress Series 140*, 285-298 (1996).

15. Toscano, M.A. and Macintyre, I.G. Corrected western Atlantic sea-level curve for the last 11,000 years based on calibrated 14C dates from Acropora palmata framework and intertidal mangrove peat. *Coral reefs*, *22*(3), 257-270 (2003).

16. Cahoon, D.R. and Lynch, J.C. Vertical accretion and shallow subsidence in a mangrove forest of southwestern Florida, USA. *Mangroves and Salt Marshes*, *1*(3), 173-186 (1997)

17. Parkinson, R.W., DeLaune, R.D. and White, J.R. Holocene sea-level rise and the fate of mangrove forests within the wider Caribbean region. *Journal of Coastal Research*, 1077-1086 (1994).

18. Toscano, M.A., Gonzalez, J.L. and Whelan, K.R. Calibrated density profiles of Caribbean mangrove peat sequences from computed tomography for assessment of peat preservation, compaction, and impacts on sea-level reconstructions. *Quaternary Research*, *89*(1), 201-222 (2018).

19. Lynch, J.C., Meriwether, J.R., McKee, B.A., Vera-Herrera, F. and Twilley, R.R. Recent accretion in mangrove ecosystems based on 137 Cs and 210 Pb. *Estuaries*, *12*(4), pp.284-299 (1989).

20. Callaway, J.C., DeLaune, R.D. and Patrick Jr, W.H. Sediment accretion rates from four coastal wetlands along the Gulf of Mexico. *Journal of Coastal Research*, 181-191 (1997).

21. McKee, K.L. Biophysical controls on accretion and elevation change in Caribbean mangrove ecosystems. *Estuarine, Coastal and Shelf Science*, *91*(4), 475-483 (2011).

22. Meeder, J.F., Parkinson, R.W., Ruiz, P.L. and Ross, M.S. Saltwater encroachment and prediction of future ecosystem response to the Anthropocene Marine Transgression, Southeast Saline Everglades, Florida. *Hydrobiologia*, *803*(1), 29-48 (2017).

23. Yao, Q. and Liu, K. Dynamics of marsh-mangrove ecotone since the mid-Holocene: a palynological study of mangrove encroachment and sea level rise in the Shark River Estuary. *PLoS One* 12(3): e0173670. doi:10.1371/journal.pone.0173670 (2017)

24. Woodroffe, C.D. Mangrove swamp stratigraphy and Holocene transgression, Grand Cayman Island, West Indies. *Marine Geology* 41, 271-294 (1981).

25. Ellison, J.C., Mangrove retreat with rising sea-level, Bermuda. *Estuarine, Coastal and Shelf Science*, *37*(1), 75-87 (1993)

26. Wooller, M.J., Morgan, R., Fowell, S., Behling, H. and Fogel, M. A multiproxy peat record of Holocene mangrove palaeoecology from Twin Cays, Belize. *The Holocene*, *17*(8), 1129-1139 (2007).
