# Supplementary material for: Rapid inundation of southern Florida coastline despite low relative sea-level rise rates during the late-Holocene
Source: Nat Commun. 2019 Jul 19;10:3231. doi: 10.1038/s41467-019-11138-4 (PMC6642092; doi:10.1038/s41467-019-11138-4)
Supplement: Supplementary file 2 — Description of Additional Supplementary Files [file 41467_2019_11138_MOESM2_ESM.pdf]

Description of Additional Supplementary Files

File Name: Supplementary Data 1
Description: Pollen data for all four cores, presented as raw counts.

File Name: Supplementary Data 2
Description: Stable isotope data for all four cores.

File Name: Supplementary Data 3
Description: Occurrence of mollusks and other invertebrate and/or calcareous taxa.
